# Dynamic structures of a membrane transporter in native cellular membranes

**DOI:** 10.1126/sciadv.adv4583

**Published:** 2025-11-12

**Authors:** Huayong Xie, Yuefang Gan, Weijing Zhao, Mojie Duan, Yang Shen, Huan Tan, Yan Zhang, Qiong Tong, Yongxiang Zhao, Jun Yang

**Affiliations:** ^1^ National Center for Magnetic Resonance in Wuhan, Key Laboratory of Magnetic Resonance in Biological Systems, State Key Laboratory of Magnetic Resonance and Atomic and Molecular Physics, Wuhan Institute of Physics and Mathematics, Innovation Academy for Precision Measurement Science and Technology, Chinese Academy of Sciences, Wuhan 430071, P. R. China.; ^2^ Interdisciplinary Institute of NMR and Molecular Sciences, School of Chemistry and Chemical Engineering, The State Key Laboratory of Refractories and Metallurgy, Wuhan University of Science and Technology, Wuhan 430081, P. R. China.; ^3^ University of Chinese Academy of Sciences, Beijing 100049, P. R. China.; ^4^ Laboratory of Chemical Physics, National Institute of Diabetes and Digestive and Kidney Diseases, National Institutes of Health, Bethesda, MD 20892-0520, USA.

## Abstract

Substrate transport through membrane transporters is a dynamic process that involves transitions between different functional conformations, which can be disrupted by non-native membrane mimetics. Capturing these conformations and their transitions within native cellular membranes presents a notable challenge. Herein, we used in situ solid-state nuclear magnetic resonance (NMR) to resolve the 1.5-Å outward-open and 2.5-Å occluded structures of *Bj*SemiSWEET within its native cellular membranes. Our findings reveal that these two conformations exchange within transmembrane helix TM1 and Loop L2-3 on a millisecond to second timescale, with the exchange rate corresponding to the sucrose transport rate. Molecular dynamics simulations further confirmed that these conformations represent functional states during sucrose transport. In contrast, we observed different conformational dynamics of *Bj*SemiSWEET in DMPC/DMPG synthetic bilayers compared to cellular membranes. This study highlights the potential of in situ solid-state NMR to provide previously unknown dynamic structural insights into cellular molecular processes, representing a substantial advancement in dynamic cellular structural biology.

## Teaser

The dynamic structures of a membrane transporter in its native cellular membranes were determined by in situ solid-state NMR.

## INTRODUCTION

Membrane transporters are essential membrane proteins that facilitate the movement of substrates and ions across biological membranes (*1*). They are crucial for maintaining cellular homeostasis, regulating signaling pathways, and transporting key nutrients, waste products, and signaling molecules (*2*). The transport process involves the conformational transitions in the transporter proteins. For instance, the “alternating access” model describes how these proteins transition to expose substrate binding sites to either side of the membrane (*3*). Characterizing the atomic-resolution structures of transporters in distinguishing functional states is vital for understanding their molecular mechanism (*1*, *4*). It is particularly important to study these structures within native cellular membrane environments, as subtle difference in conformations can arise due to the surrounding lipid composition (*5*, *6*). However, characterizing functional states of transporters in native environments poses notable challenges to general structural biology techniques (*1*, *7*).

In recent years, structural biology techniques, such as single-particle cryo–electron microscopy (cryo-EM) and x-ray crystallography, have captured high-resolution three-dimensional (3D) structures of transporter, revealing distinct conformations in detergent environments (*1*). These structures are typically stabilized by external interactions, including the use of antigen-binding fragments (Fabs) (*8*), nanobodies (Nbs) (*9*), fusion tags (*10*), symmetry scaffolds (*11*), small-molecule ligands (*12*), and internal interactions from engineered mutants (*3*).

The transport of substrate is a dynamic process involving transition between different functional states (*1*, *13*). Characterizing this transition is critical for understanding the functional mechanism of transport proteins (*4*, *14*–*18*). However, techniques like x-ray crystallography and cryo-EM struggle to monitor these dynamic changes (*14*, *19*). Instead, molecular dynamics (MD) simulations were used to reproduce transitions between functional states based on experimental structures. While these approaches provide insights into the alternating access mechanism of membrane transport proteins (*3*), structures determined in in vitro environments may be influenced by non-native conditions (*5*, *20*–*22*). Factors such as membrane (detergent) composition, hydrophobicity, and curvature can alter transporter structures in different functional states (*5*, *6*, *23*). Additionally, methods used for stabilizing functional states, such as Nbs, fusions, and ligands, may disturb the native structure of transporters (*1*, *24*). These disturbances can lead to misinterpretations of their functional mechanism based on simulations (*20*, *22*, *25*).

In situ solid-state nuclear magnetic resonance (ssNMR) allows for the characterization of membrane protein structure and dynamics in their native cellular membrane environments (*7*, *26*–*36*). We have developed an in situ ssNMR method, enabling structure determination of membrane proteins in *Escherichia coli* cellular membranes (*26*, *37*). This study focuses on functional mechanism of the “minimal” membrane transport protein, *Bradyrhizobium japonicum* SemiSWEET (*Bj*SemiSWEET) (*38*, *39*), which consists of three transmembrane helices (TM1, TM2, and TM3) that form dimers to transport sucrose (*4*). While 3D structures of SemiSWEETs in various functional states (outward-open, inward-open, and occluded) have been determined by x-ray crystallography in detergent environments (*3*, *4*, *39*–*41*), these structures may be influenced by their non-native conditions (*5*, *6*, *23*). To obtain native structure and accurate molecular mechanism of SemiSWEET, we used in situ ssNMR to determine the high-resolution structures of the outward-open and occluded conformations of *Bj*SemiSWEET in its native cellular membranes. We observed exchanges between these two conformations of the protein on the millisecond to subsecond timescale. MD simulations suggested that these two conformations represent functional states during sucrose transport. Our findings highlight importance of studying membrane proteins in in situ environment, showcasing the potential of in situ ssNMR for elucidating their structural mechanism in native cellular membrane environment.

## RESULTS

### Two distinct conformations of *Bj*SemiSWEET were detected in the cellular membrane, while only one conformation was observed in synthetic lipid bilayers

**Fig. 1.**
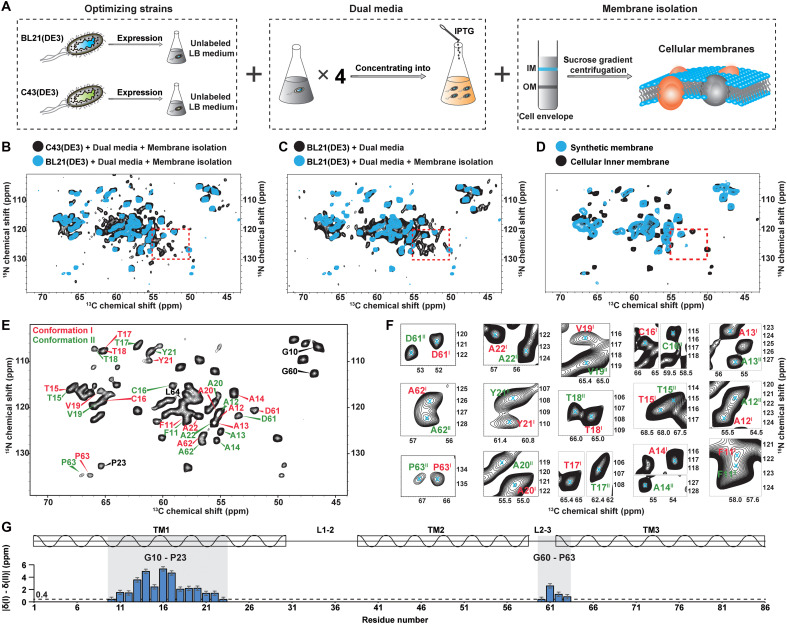
Two distinct conformations of *Bj*SemiSWEET in cellular membranes were detected by in situ ssNMR. (**A**) Schematic diagram of the preparation process for *Bj*SemiSWEET cellular membrane samples. Previous studies have shown the importance of selecting the appropriate bacterial strain for successful native membrane sample preparation. After screening various strains, B21(DE3) strain was identified as the optimal choice for expressing the target protein at high level with minimal background protein expression. The strain was cultured in natural abundance LB medium and then concentrated 4:1 into ^13^C, ^15^N–labeled M9 medium (dual media). The outer membrane components were separated by sucrose density gradient centrifugation, and the inner membrane of *E. coli* was collected. (**B**) Overlay of 2D NCA spectra from *Bj*SemiSWEET cellular membrane samples derived from different expression strains. The cyan spectrum corresponds to strain BL21(DE3), and the black spectrum represents strain C43(DE3). The red boxes highlight regions containing background proteins, indicating that the BL21(DE3) strain has reduced background protein levels. (**C**) Overlay of 2D NCA spectra from different cellular membranes of *Bj*SemiSWEET. The cyan spectrum represents the inner membrane, and the black spectrum represents the total membrane. The red box indicates that membrane separation effectively decreased background protein signals. (**D**) NCA spectra of cellular membranes (cyan) overlaid with synthetic membranes (DMPC/DMPG) (black). The red box indicates an absence of background protein signal in the cellular membrane sample. (**E**) The 2D NCA spectra of *Bj*SemiSWEET in cellular membranes illustrate the assignments of 15 residues with two distinct sets of chemical shifts. Assignments for conformation I are depicted in red, while those for conformation II are shown in green. (**F**) Close-up views of the 2D NCA spectra emphasize the differences in chemical shifts among the 15 residues. (**G**) Discrepancies in chemical shifts for the two conformations of *Bj*SemiSWEET.

To characterize the structure of *Bj*SemiSWEET in native cellular membranes, we prepared cellular membrane samples for ssNMR experiments. For comparison, we also prepared two samples of the purified *Bj*SemiSWEET reconstituted into 1,2-dimyristoyl-sn-glycero-3-phosphocholine/1,2-dimyristoyl-sn-glycero-3-phospho-(1′-rac-glycerol) (DMPC/DMPG) and 2-oleoyl-1-pamlitoyl-sn-glyecro-3-phosphocholine/2-oleoyl-1-pamlitoyl-sn-glyecro-3-glycerol (POPC/POPG) lipid bilayers, which differ in hydrophobic thickness due to their acyl chain lengths (14 for DMPC/DMPG and 16/18 for POPC/POPG). In our previous study (*26*, *37*), we developed a cellular membrane sample preparation protocol that enables high-quality ssNMR spectra without the interference of background protein signals. This protocol uses three strategies to enhance target protein density and suppress background protein expression and isotopic labeling in cellular membrane (Fig. 1A): optimizing protein expression strains, using dual media for protein expression, and isolating membrane components. The *E. coli* inner membrane samples from the BL21(DE3) expression strain yielded cleaner spectra than those from the C43(DE3) strain, enhancing spectral sensitivity and nearly eliminating outer membrane background signals (Fig. 1, B and C). The additional signals in cellular membranes, compared to purified *Bj*SemiSWEET in synthetic membranes, originate from *Bj*SemiSWEET itself (Fig. 1D and fig. S1A). Peak shifts in the spectra indicate conformational variations of the protein between the two environments. We successfully assigned chemical shifts for 81 of 86 residues, because of the high-quality spectra with minimal background interference (fig. S1A).

During the assignment process, we unexpectedly identified two sets of chemical shifts for residues G10-P23 and G60-P63 (Fig. 1, E to G) in *Bj*SemiSWEET. The representative backbone walks for G60 to P63 in the two conformations (I and II) are shown in fig. S1B. In these spectra, the C′ of G60 displayed a single chemical shift of 173.8 parts per million (ppm). By fixing the C′ of G60 at this value in the 3D NCOCX and CONCA spectra, we then assigned two sets of ^15^N and ^13^Cα chemical shifts for D61: D61(I) (120.5, 52.0 ppm) and D61(II) (121.8, 53.5 ppm), which were confirmed by the 2D NCA spectra (Fig. 1, E and F). Using the chemical shifts of D61(I) and D61(II), we then assigned the shifts for A62(I), P63(I), A62(II), and P63(II) through the backbone walk. The two conformations of A62 and P63 residues corresponded to distinguish peaks in the 2D NCA spectra (Fig. 1, E and F).

The secondary structures of both conformations, predicted from their assigned chemical shifts, consist of three transmembrane α-helices (fig. S1C). Although residues G10-P23 in TM1 and G60-P63 in the loop between TM2 and TM3 (Loop L2-3) show different chemical shifts (Fig. 1G), their secondary structures remain similar (fig. S1C).

To explicitly rule out potential artifacts introduced by sucrose gradients during the preparation of inner membranes, we compared NCA spectra from sucrose-depleted (total membrane) and sucrose-exposed (inner membrane) samples. Consistent dual conformational states were observed for residue D61 even in sucrose-free total membrane preparations (fig. S2, A and B). To further examine whether sucrose binding artificially generates additional conformations, we introduced a large excess of sucrose (molar ratio 1:10,000) to *Bj*SemiSWEET proteoliposomes. The NCA spectra collected before (red) and after (green) sucrose addition are nearly identical. No new conformational state appeared for residue D61 upon sucrose binding (fig. S2, C and D). Together, these results provide strong evidence that the conformational states observed in inner membranes are intrinsic to *Bj*SemiSWEET and not artifactual effects of residual sucrose.

**Fig. 2.**
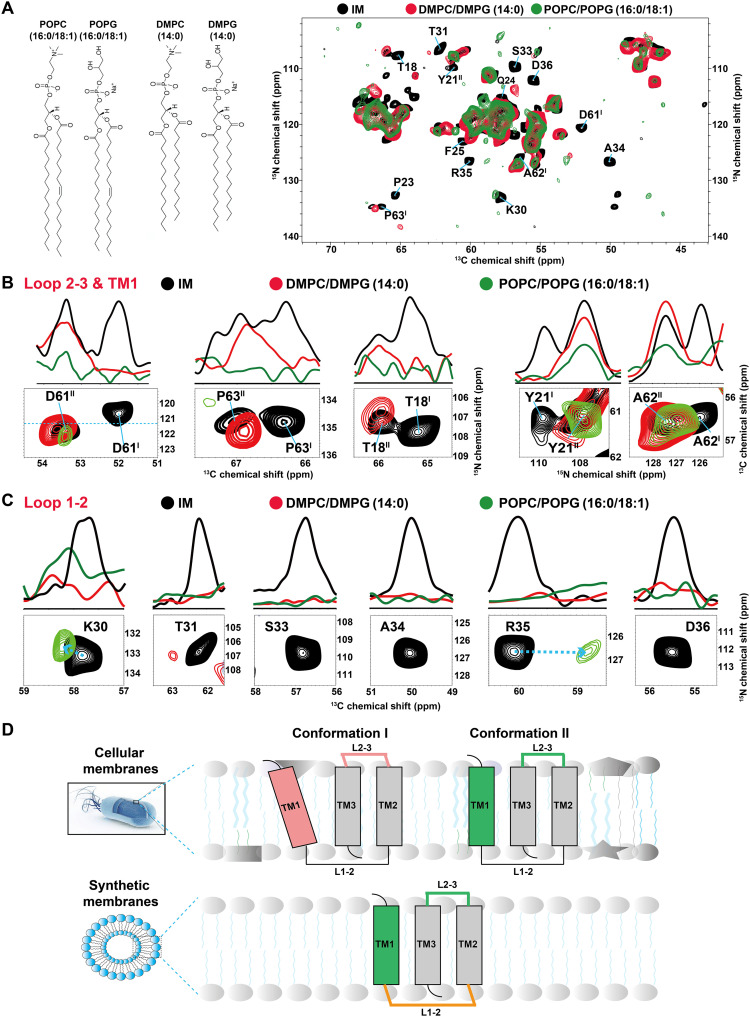
The ssNMR spectra of *Bj*SemiSWEET in cellular membranes differ from those in synthetic lipid bilayers. (**A**) Overlay of 2D NCA spectra of *Bj*SemiSWEET in the cellular membrane (in black), 14:0 DMPC/DMPG (in red), and 16/18:0 POPC/POPG (in green), with structural formulas of the four phospholipids displayed on the left. (**B**) Sections of the NCA spectrum display residues in the Loop L2-3 and TM1 regions of *Bj*SemiSWEET exhibiting two conformations in the cellular membranes and a single conformation in each distinct synthetic lipid bilayers. The top 1D cross sections show projections calculated over the chemical shift regions depicted in the corresponding spectra. (**C**) Sections of the NCA spectrum exhibit residues in the Loop L1-2 region of *Bj*SemiSWEET with varying spectral sensitivity in the cellular membranes compared to various synthetic membranes. (**D**) A schematic depiction of the conformation of *Bj*SemiSWEET in cellular and synthetic membranes reveals that the Loop L2-3 and TM1 regions exhibit two conformations in cellular membranes and one conformation in synthetic membranes. Moreover, the intracellular Loop L1-2 displays distinct conformation dynamics in these distinct membrane environments.

In contrast, all residues of *Bj*SemiSWEET in DMPC/DMPG and POPC/POPG bilayers, including residues G60-P63 and P10-G23, exhibit a single set of chemical shifts, indicating only one conformation (Fig. 2, A and B). The chemical shifts of G60-P63 and P10-G23 in synthetic lipid bilayers resemble those in conformation II of *Bj*SemiSWEET in cellular membranes. However, there are differences in chemical shifts or spectral sensitivities of residues in Loop L1-2, reflecting different conformational states and dynamics in this region (Fig. 2, C and D). These structural differences suggest potential functional consequences. Consistent with this, fluorescence resonance energy transfer (FRET)–based sucrose transport assays confirmed that *Bj*SemiSWEET reconstituted into DMPC/DMPG liposomes exhibited no detectable transport activity (fig. S3), showing no substantial difference in uptake rates compared to the protein-free control. This lack of detectable activity could stem from two possibilities: (i) an inherent inability of the DMPC/DMPG lipid composition to support functionality, or (ii) protein activity in DMPC/DMPG that is below the detection limit of the assay. Therefore, the *Bj*SemiSWEET in DMPC/DMPG bilayers used for structural comparison is nonfunctional under these assay conditions.

To evaluate the potential influence of lipid-to-protein ratios, we quantified this parameter in both native cellular membranes and synthetic DMPC/DMPG liposomes (table S1). Despite a marginally lower mass ratio in synthetic vesicles [1.25:1 (w/w) versus 1.4:1 (w/w) in native membranes], molar ratios were comparable (~16:1 mol/mol), indicating no notable stoichiometric divergence between systems. To test whether lipid-to-protein ratio modulates conformational states, we acquired 1D ^15^N spectra of *Bj*SemiSWEET in synthetic vesicles across a ratio range from 1.0 to 2.5 (w/w; fig. S4, A to F). Strikingly, spectral patterns remained nearly identical across all synthetic preparations but differed markedly from those in native membranes, roughly suggesting the presence of only a single conformational state in all synthetic vesicle samples though without residue-specific details. To provide residue-specific details, we prepared a uniformly ^13^C, ^15^N–labeled *Bj*SemiSWEET sample in DMPC/DMPG liposomes at lipid-to-protein ratio = 2 and acquired a 2D NCA spectrum. The spectrum confirms the presence of only a single set of correlations for key residues such as D61 and A62 (fig. S4, G and H), consistent with a single predominant conformation.

Conformational changes in TM1 and Loop L2-3 of SemiSWEET in two conformations are associated with the gating of the outer gate (*3*, *41*). To elucidate the functional relevance of these conformations, we determined the 3D structures of these two conformations of *Bj*SemiSWEET in cellular membranes.

### High-resolution 3D structures of the outward-open and occluded conformations of *Bj*SemiSWEET in cellular membrane were determined by in situ ssNMR

To obtain sufficient distance restraints for structure determination, we acquired 2D ^13^C-^13^C chemical shift correlation spectra using different recoupling methods and sample labeling schemes (table S2). Two 2D ^13^C-^13^C COmbined $R2_{n}^{v}$-Driven (CORD) spectra (*42*) with a 500-ms mixing time of ^13^C sparsely labeled *Bj*SemiSWEET, expressed using 2-^13^C- and 1,3-^13^C-glycerol as carbon source (*43*), provided the majority of distance restraints. Additionally, proton-assisted recoupling (PAR) spectra (*44*) with a 20-ms mixing time and CHHC spectra (*45*) with a 500-μs mixing time of uniformly ^13^C, ^15^N–labeled *Bj*SemiSWEET contributed supplementary distance restraints (table S3). All these spectra exhibited serious ambiguity in assigning ^13^C-^13^C distance restraints, even in the sparse labeling spectra (fig. S5 and table S4). To mitigate this ambiguity, we used structural models predicted by CS-Rosetta, which incorporated all assigned chemical shifts and 16 initial ambiguous distance restraints (table S4). The resulting structural models achieved a root mean square deviation (RMSD) value of 2.1 and 3.5 Å, respectively, for the converged lowest energy models, owing to nearly complete chemical shift assignments (fig. S6). It is important to note that these calculations generated converged structural models only when performed in membrane environments and based on the initial distance restraints.

**Fig. 3.**
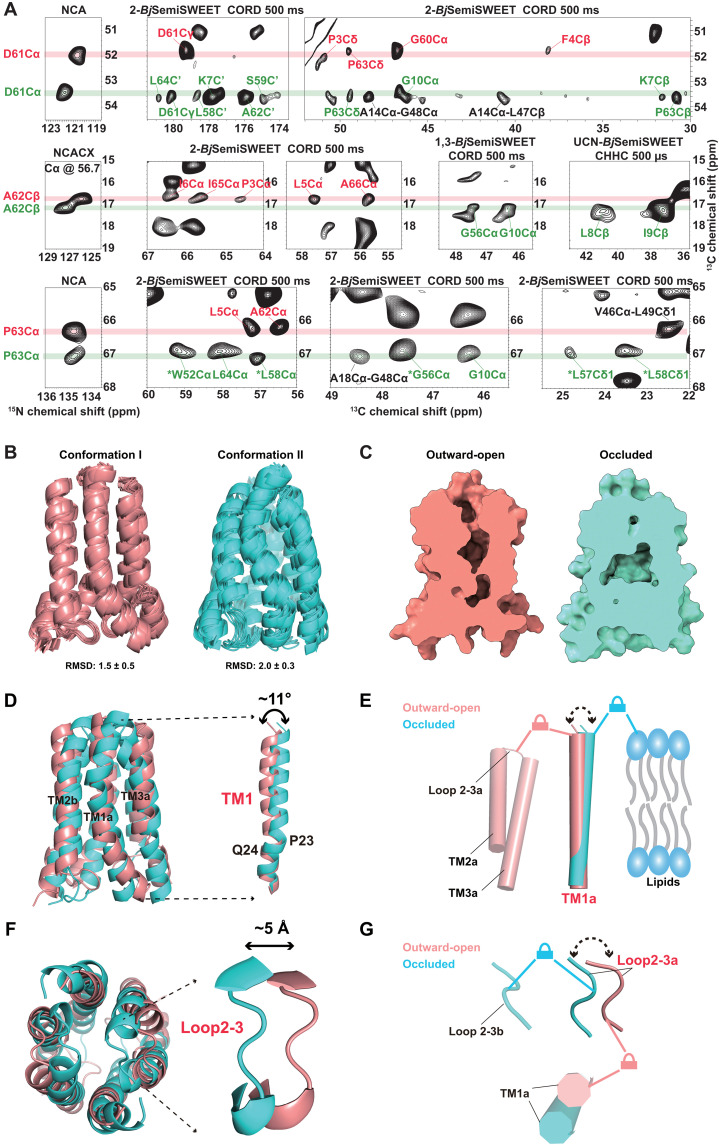
In situ ssNMR structures of *Bj*SemiSWEET reveal outward-open and occluded states within cellular membranes. (**A**) Sections in the ssNMR spectra of *Bj*SemiSWEET display signals corresponding to varying distance restraints of residues D61, A62, and P63 in two conformations (I: red; II: green). Dashed lines indicate peaks aligned with potential distance restraints. Signals in red and green fonts represent distance constraints for conformation I and II, respectively, while signals in black font denote distance restraints unrelated to residues D61, A62, and P63. (**B**) Superimposition of the 10 lowest energy dimer structures representing the two conformations of *Bj*SemiSWEET. Conformation I and II are depicted in pink and cyan, with backbone RMSD values of 1.5 ± 0.5 Å and 2.5 ± 0.3 Å, respectively. (**C**) Slab views: I (pink) shows outward-open state; II (cyan) shows occluded state. (**D**) Superimposition highlights TM1 helix angle difference. (**E**) Mechanisms for stabilizing TM1 in two conformations. In conformation I, TM1 (red) is upheld by interactions between TM1 residues and Loop L2-3 residues within the same monomer, whereas in conformation II, TM1 (cyan) is supported by electrostatic interactions between TM1 residues and lipid headgroups. (**F**) Superimposition reveals Loop L2-3 positional shift. (**G**) Mechanisms for stabilizing Loop L2-3 in the two conformations. In conformation I, Loop L2-3 (red) is maintained by interactions between Loop L2-3 residues and TM1 residues within the same monomer, whereas in conformation II, Loop L2-3 (cyan) is reinforced by interactions between Loop L2-3 residues and Loop L2-3 residues from adjacent monomer.

It is notable that residues with different chemical shifts in the two conformations correspond to distinct distance restraints. As shown in Fig. 3A, in conformation I, D61 is associated with two long-range distance constraints: D61(I)Cɑ-P3Cδ and D61(I)Cɑ-F4Cβ, while in conformation II, D61 does not show these signals. Instead, D61 in conformation II relates to three different long-range distance constraints: D61(II)Cɑ-K7C′, D61(II)Cɑ-G10Cɑ, and D61(II)Cɑ-K7Cβ, which are absent in conformation I. Overall, residues D61, A62, and P63 contribute to 6 long-range distance constraints in conformation I and 13 in conformation II. As shown in fig. S7 and table S5, all six long-range distance constraints in conformation 1 are intramolecular, while conformation II includes eight intramolecular long-range distance constraints and five intermolecular long-range distance constraints (P63Cɑ-W52Cɑ, P63Cɑ-L56Cɑ, P63Cɑ-L57Cγ1, P63Cɑ-L58Cɑ, and P63Cɑ-L58Cδ1). These differing distance constraints indicate substantial structural variations in the corresponding local regions between the two conformations.

The dimeric arrangement epitomizes an evolutionarily conserved structural characteristic within the SemiSWEET family, as extensively illustrated by Feng *et al.* (*4*, *40*). Direct determination of oligomeric state of *Bj*SemiSWEET in native membranes remains technically challenging. The inherent interfacial resilience of *Bj*SemiSWEET dimers, as evidenced by their persistence in both detergent (fig. S8A) (*40*) and liposomal settings (fig. S8B), fortifies their prevalence within the native membrane environment. We incorporated the distance constraints (fig. S7), along with 162 angle constraints predicted by TALOS+ (*46*) and 74 hydrogen bonds identified by HBPLUS (*47*), into calculations by Xplor-NIH (*48*). This process generated 1000 structures for each conformation. The backbone RMSD of the top 10 energetically minimized dimeric structures among these calculated structures was 1.5 ± 0.2 Å for conformation I and 2.5 ± 0.3 Å for conformation II (Fig. 3B and table S6).

Like other resolved SemiSWEET structures, the monomer of *Bj*SemiSWEET in both conformations consists of three transmembrane helices, with TM3 positioned between TM1 and TM2 (Fig. 3B) (*3*, *4*). During substrate transport, SemiSWEET adopts various conformations, including outward-open, inward-open, and occluded states. To identify open states of these two conformations, we quantitatively analyzed their pore shapes using the MOLE software (fig. S9, A to D) (*49*). Conformation I features a cone-shaped opening extending up to 20 Å toward the extracellular side, indicating an outward-open conformation (Fig. 3C and fig. S9, A and B). In contrast, conformation II has a large cavity defined by residues W52, N68, and Y21, indicating an occluded conformation (Fig. 3C and fig. S9, C and D).

The open and closed states of *Bj*SemiSWEET were validated by H/D exchange experiments. The inner surface of the pore in the open conformation is more accessible, resulting in higher level of H/D exchange. As shown in fig. S10, both conformations exhibit similar low proton preservation in Loop L2-3, with ~40% and ~80% proton preservation in TM1 for conformation I and II, respectively. These results are consistent with their outward-open and closed states in cellular membrane.

What structural differences between two conformations lead to their different states? On the basis of the experimentally determined structures of the two *Bj*SemiSWEET conformations (Fig. 3, B and C), notable variations are observed in TM1 and Loop L2-3 of the dimeric structures of the two conformations (fig. S9, E and F). The TM1 helix in conformation I has a tilt angle of ~11° compared with TM1 in conformation II (fig. S9E and Fig. 3D), and the backbone RMSD of residues in Loop L2-3 is ~5 Å between the two conformations (fig. S9F and Fig. 3F). These differences align with the chemical shift variations of these residues (Fig. 1G). To further analyze the interactions stabilizing the different local conformations, we performed all-atom MD simulations with enhanced sampling technology to characterize the important residue-residue and residue-lipid interactions in the occluded and outward-open structures (fig. S11). The MD simulations results revealed that TM1 and Loop L2-3 in the outward-open structures are stabilized by intra-monomeric interactions between them (Fig. 3E and fig. S11B). In contrast, the MD simulations results of occluded structures indicated that TM1 is stabilized by electrostatic interactions with lipid headgroups, which weakens the intra-monomeric interactions with Loop L2-3 (fig. S11C). This release allows Loop L2-3 to interact with inter-monomeric Loop L2-3 residues, resulting in an occluded conformation (Fig. 3G and fig. S11A). The stabilization of specific conformations by lipid-protein interactions is also observed in many other transport proteins (*6*, *23*). A more detailed analysis of stabilizing interactions is provided in fig. S11.

### Exchange of two conformations of *Bj*SemiSWEET in cellular membrane was detected by in situ ssNMR

**Fig. 4.**
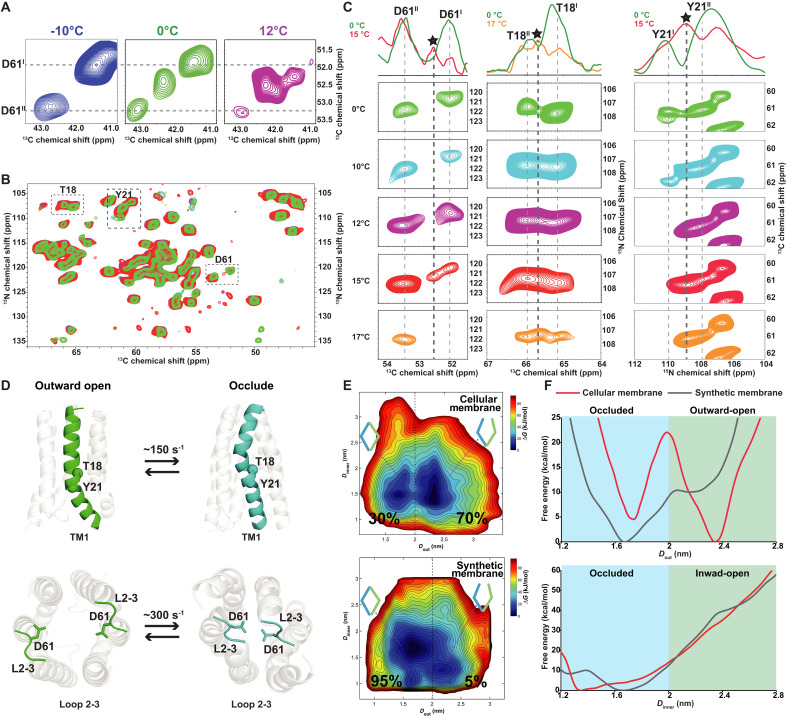
Conformational exchange of *Bj*SemiSWEET in cellular membranes monitored by temperature-dependent ssNMR spectra. (**A**) The averaged signals of the Cɑ-Cβ correlation for residue D61 are present in both conformations in the 50-ms ^13^C-^13^C spectrum of *Bj*SemiSWEET in cellular membranes acquired at 273 and 285 K, while absent at 263 K. (**B**) Superimposition of 2D NCA spectra at five different temperatures: 273 K (green), 283 K (cyan), 285 K (purple), 288 K (red), and 290 K (orange). Figure S12 displays each spectrum separately. (**C**) Presence of 2D exchange peaks for the N-CA correlation peaks of residues D61, T18, and Y21 in both conformations at 288 K and above, with an absence in the spectra at 273 K. The top 1D projections illustrate exchange cross peaks between conformation I and II. The projection ranges are as follows: D61 (119.0 to 124.0 ppm), T18 (105.5 to 109.0 ppm) and Y21 (59.5 to 62.5 ppm). (**D**) Exchanging residues: T18/Y21 on TM1; D61 on Loop L2-3. (**E**) 2D free energy landscapes (intra-/extracellular gate distances via D61-D36 Cα): Synthetic membranes (DMPC/DMPG): 95% occluded, 5% outward-open. Cellular membranes: 30% occluded, 70% outward-open. (**F**) 1D conformational free energy landscape of *Bj*SemiSWEET in the native cellular membrane and synthetic membrane (DMPC/DMPG). The energy difference between the two conformations is minor in the native cellular membrane (approximately 5 kJ/mol). However, in the synthetic membrane, the major conformation is the outward-open conformation, and the energy difference between the two conformations is larger (~10 kJ/mol).

SemiSWEET transitions between different conformational states during sucrose transport, as revealed by MD simulations. Can this transition be captured experimentally? We observed that the Cɑ-Cβ correlation peaks of residue D61 in the two conformations exhibit averaged chemical shifts in the 2D ^13^C-^13^C correlation spectrum of *Bj*SemiSWEET at 273 K, along with previously unknown averaged peaks (52.8, 42.2 ppm) (Fig. 4A). This suggests the possible exchange between the two conformations on an intermediate timescale (*17*, *50*), although general correlated signals (i.e., cross peaks due to magnetization transfer) cannot be excluded. To distinguish whether these averaged peaks result from conformational exchange or general correlation, we conducted ssNMR experiments at varying temperatures. Lowering the temperature weakened the exchange peaks due to “freezing” the exchange, while general correlation signals strengthened, as dipolar couplings are enhanced at lower temperatures. Figure 4A shows the disappearance of the averaged peak at 263 K and its enhancements at 285 K, confirming that the signal derived from conformational exchange rather than general correlations. Similar temperature-dependent exchange patterns were observed for TM1 residues T18 and Y21 in 2D NCA spectra (Fig. 4, B to D, and fig. S12). Crucially, cyclic temperature experiments confirmed full reversibility of these spectral changes (fig. S13). The temperature-dependent changes in spectral line shapes are consistent with a modulation of the exchange rate between these two states (Fig. 4D). This behavior follows an Arrhenius-type temperature dependence [*k*(*T*) = *k*_0_ exp(−*E*_a_/*RT*)], supporting the validity of the two-site model in capturing the essential dynamic features across temperatures. Furthermore, 2D CEST-NCA and 2D CSA-CODEX-NCA experiments provide direct evidence of conformational exchange in *Bj*SemiSWEET (fig. S14). An exchange rate (*k*_ex_) of approximately 56.2 s^−1^ was observed for residue D61 (fig. S15), classifying it within the intermediate exchange regime (Δω = 104 s^−1^, *k*_ex_/Δω ~ 0.54) (*51*).

Why does *Bj*SemiSWEET exhibit two exchangeable conformations in cellular membranes but not in synthetic lipid bilayers? To investigate the conformational landscape of *Bj*SemiSWEET in different membrane environments, we conducted accelerated sampling by metadynamics simulations (Fig. 4E). The results showed that in cellular membranes, *Bj*SemiSWEET exists in occluded and outward-open conformation with populations of 30% and 70%, respectively, and a low energy difference of ~5 kJ/mol between them (Fig. 4F). This low energy difference aligns with the observed exchangeable conformations in ssNMR experiments. In contrast, in DMPC/DMPG bilayers, the population was 95% for occluded and 5% for outward-open conformations, with a higher energy difference of ~10 kJ/mol. This supports the observation of a single predominant conformation of *Bj*SemiSWEET in lipid bilayers (Fig. 4E).

### Two conformations of *Bj*SemiSWEET in native cellular membranes are functional states in sucrose transport

**Fig. 5.**
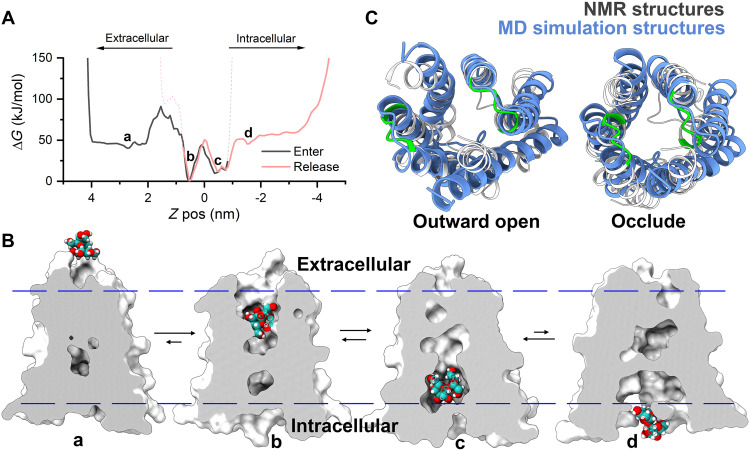
The two distinct conformations of *Bj*SemiSWEET observed in the cellular membrane represent functional states in sucrose transport as revealed by MD simulations. (**A**) Free energy profiles of sucrose entry and release processes, with the vertical axis representing the reaction activation energy and the horizontal axis representing the distance from sucrose to the center of the channel along the *z* axis. Depending on the value of the activation energy, the process is divided into four stages (a to d). (**B**) Typical structures of *Bj*SemiSWEET at the four stages of the sucrose transport process, where stages a and c denote the occluded conformation, while stages b and d represent the outward-open and the inward-open conformation, respectively. (**C**) The structures of the outward-open and the occluded conformations in the sucrose transport process in MD simulations align with the ssNMR structures, exhibiting RMSDs of 2.4 Å and 3.1 Å, respectively. This alignment indicates that the ssNMR structures correspond to these functional states.

To investigate the functional relevance of the two conformations, we performed Funnel metadynamics (FM) simulations to study the transporting processes of sucrose through *Bj*SemiSWEET, starting with the experimentally determined outward-open structure (Fig. 3B). To better characterize the whole transporting process, we divided the sucrose transport into the entry and the release subprocesses, in which the sucrose is limited in the confined spaces by the restraint potential walls (fig. S16). Both the entry and release process were described by two reaction coordinates, including the positions of the ligand and the degree of opening of the entry/release gates (Fig. 5).

As shown in Fig. 5A, the activation energies for the two processes support a unified description of the complete sucrose transport process. We categorized the transport process into four stages: Stages a and c represent the occluded conformations, stage b represents the outward-open conformation, and stage d represents the inward-open conformation (Fig. 5B). Notably, only the inward-open conformation (d) was not captured by ssNMR, likely due to its high free energy (Fig. 4F). The remaining three conformations aligned well with the ssNMR structures, indicating that the ssNMR structures correspond to these functional states (Fig. 5C). Previous studies have suggested that substrate transport occurs via a “free ride” mechanism; thus, the observed interconversion between two conformations can be related to sucrose transport. Furthermore, previous functional experiments have shown that the rate of sucrose transport by *Bj*SemiSWEET is in the millisecond to second range (*38*), consistent with the exchange rates observed in ssNMR experiments.

## DISCUSSION

Here, advancements in in situ ssNMR enabled determination of the structure of a membrane protein with unknown structure in native cellular membranes, capturing transitions between different functional states, contribute to understanding of the undisturbed molecular mechanism. We demonstrate the feasibility and potential of in situ ssNMR for simultaneously determining the atomic-resolution structure and revealing conformational dynamics of membrane transport proteins within the cellular membrane environments.

In situ ssNMR offers unique advantages in elucidating the structural mechanisms of membrane proteins (*7*, *26*–*29*). First, it enables the determination of high-resolution 3D structures in the native cellular membrane environment (*26*). Cellular membranes are complex matrix primarily composed of lipids, proteins, and carbohydrates, featuring over 1000 lipid varieties with diverse head groups, acyl chain lengths (14 to 18 carbons), and varying saturation levels (*52*). Additionally, the lipid bilayer structure of cellular membranes is asymmetrical in the lipid compositions in the upper and lower leaflets, influencing the orientation of membrane proteins to the membrane. The composition and physicochemical properties of cellular membranes such as hydrophobicity, curvature, and gradients can substantially affect the structure and function of membrane proteins, particularly membrane-sensitive transporters (*25*). Not all conformations of *Bj*SemiSWEET can be observed in synthetic lipid bilayers, likely due to differences between these and cellular membranes. Second, in situ NMR allows for the study of membrane transporters under the native conditions, including physiological temperature and wild-type sequences, without external stabilizing conditions that may disturb protein structure. This capability facilitates the observation of native structures and conformational exchanges relevant to substrate transport. In contrast, low temperatures in x-ray crystallography and cryo-EM hinder the observation of dynamic processes that occur at physiological temperatures (*14*–*16*, *19*). Finally, in situ ssNMR can monitor transitions between distinct conformational states at physiological temperatures, which is crucial for exploring molecular mechanisms (*4*, *15*, *16*, *19*). The dynamic details captured by in situ NMR are often unavailable using other structural techniques (*7*, *14*).

The advantage of in situ ssNMR is exemplified in elucidating the functional role of Loop L2-3 in *Bj*SemiSWEET. Although loops in proteins are crucial for membrane protein function, they often remain “invisible” in x-ray crystallography due to their dynamic nature (*53*). Previous studies on SemiSWEET identified the functional significance of Loop L2-3 through mutations; for instance, mutating the positively charged residue R57 in *E. coli* SemiSWEET (*Ec*SemiSWEET) increased sucrose transport activity (*41*). Similarly, mutating the negatively charged residue D57 to alanine in *Leptospira biflexa* SemiSWEET (*Lb*SemiSWEET) led to a notable rise in glucose transport activity (*3*). However, the involvement of these residues in conformational transitions was not revealed due to the limitations of earlier techniques (*3*, *4*, *39*–*41*). Here, we used in situ ssNMR to directly observe the participation of Loop L2-3 in the conformational transitions of SemiSWEET(Fig. 4, A to C). The native cellular membrane environment facilitated the observation of functional states with subtle differences, while conditions such as physiological temperature, wild-type constructs, and the absence of external stabilizers allowed for direct observation of conformational exchanges in two functional states. Our findings clarify the molecular mechanisms behind conformational transitions in membrane transporters and highlight the essential yet often overlooked role of loops.

We also observed the participation of TM1 in the conformational transitions of SemiSWEET. While the functional role of TM1 residues have been identified, their involvement in conformational transition had not been previously documented (*4*, *40*, *41*). Through chemical shift assignment, we identified the coexistence of two distinct conformations of the 12 residues of TM1 (Fig. 1F). On the basis of structures derived from distance constraints, we determined that these two conformations represent the outward-open and occluded states (Fig. 3, B and C). Furthermore, we detected the exchange of residues Y21 and T18 on TM1 between the two conformations (Fig. 4, C and D). Our study also revealed that the conserved PQ-loop motif in the TM1 helix serves as a flexible hinge, facilitating conformational transitions in SemiSWEET (*41*).

On the basis of our experimental observations and MD simulations, we propose a molecular mechanism for the conformational transitions of SemiSWEET in the native cellular membrane. When the TM1 of a monomer contacts stably with Loop L2-3, the structure stabilizes in the outward-open conformation (fig. S17B). Conversely, when the Loop L2-3 regions between monomers interact, the structure stabilizes in the occluded conformation (fig. S17A). The driving force behind these conformational transitions is the competition between the unfavorable conformation of TM1 (*3*) and the electrostatic interactions with the headgroups of phospholipids (fig. S17C). This mechanism differs from those proposed in the previous studies (fig. S17). Lee *et al.* suggested that each protomer in the *Ec*SemiSWEET conformational change undergoes a hinge-like motion, with a conserved PQ-loop acting as a hinge, leading the closure of the extracellular gate via TM1 movement (*41*). In contrast, Feng *et al.* proposed that the conformational changes in *Lb*SemiSWEET are mediated by rigid-body movements of each protomer, with the movement of TM3 facilitating the closure of the extracellular gate (*3*, *40*). The difference in mechanisms may stem from variations between species of SemiSWEET(*Ec*, *Lb*, and *Bj*) and from the fact that previous mechanisms were derived from in vitro experiments, which may have been influenced by detergent environments and methods used for stabilizing various functional states.

We determined the structures of two membrane proteins, aquaporin Z (AqpZ) (*26*) and *Bj*SemiSWEET, in cellular membrane using in situ ssNMR. Structures of two proteins exhibit different sensitivities to their membrane environments. The AqpZ’s structure remains stable even in detergent environments, owing to the tight packing of its tetrameric form and relatively minimal contact between its transmembrane domains and membrane (or mimic) (*26*). Similarly, the structure of *Anabaena* sensory rhodopsin (ASR) also shows membrane insensitivity, likely due to its compact quaternary arrangement (*33*). The well-defined tertiary and quaternary structures of AqpZ and ASR appear to be crucial for maintaining their native conformations, rather than specific protein-lipid interactions (*26*, *54*). In contrast, *Bj*SemiSWEET is highly sensitive to the membrane environment, likely due to its loose packing and extensive protein-lipid contacts. Without a stabilizing quaternary structure, *Bj*SemiSWEET’s conformation are more susceptible to perturbations from lipid bilayer (Fig. 3E and fig. S11C). For membrane proteins with flexible or loosely packed structures, like *Bj*SemiSWEET, the choice of membrane environment is critical to preserving native conformations, as lipid composition, bilayer thickness, and other properties can substantially influence protein structure and function (*20*, *25*). This finding is specific to the DMPC/DMPG reconstitution system and cannot be generalized to all synthetic lipid environments. Reconstituting *Bj*SemiSWEET in membranes that better mimic the complexity of native lipid composition possibly restores functionality and yields kinetics more closely resembling those under physiological conditions.

While lipid bilayers are often regarded as native-like in structural characterization of membrane proteins (*20*, *25*), we show that the complexity and the compositional diversity of cellular membranes are critical for preserving the native structures of membrane-sensitive membrane proteins like *Bj*SemiSWEET. By combining in situ ssNMR with other techniques, such as hydrogen-deuterium exchange mass spectrometry (HDX-MS) (*23*), cellular double electron-electron resonance (DEER) (*55*), and cellular cryo–electron tomography (cryo-ET) (*56*), we can achieve unprecedented dynamic structural insights into molecular processes occurring in live cells.

## MATERIALS AND METHODS

### Expression and purification of *Bj*SemiSWEET

*Bj*SemiSWEET, featuring an N-terminal His6-tag sequence, was expressed in *E. coli* BL21 (DE3) using a “dual media” method (*37*). Initially, the target gene with a C-terminal 6×His tag was inserted into the PET-21a vector and subsequently introduced into *E. coli* BL21 cells for expression. The bacterial culture attained an OD600 (optical density at 600 nm) of 1.1 to 1.2 before being harvested via centrifugation. Following suspension in a 4:1 ratio of M9 medium (2 g/liter ^13^C glucose, 1 g/liter ^15^N NH_4_Cl), the cells were incubated for 15 min at 37°C and induced with 1 mM isopropyl-β-d-thiogalactopyranoside (IPTG). After a 5-hour expression period at 37°C, cells were harvested, resuspended in lysis buffer (20 mM tris, 100 mM NaCl, pH 8.0), and lysed via ultrasonication. The lysate was centrifuged at 4629*g* for 10 min to eliminate unbroken cells and larger debris. The total cell membranes were then collected at 58,545*g* for 1 hour and subsequently solubilized overnight in 1% (w/v) N-lauroylsarcosinate (NLS) detergent at 4°C. This solubilized mixture underwent purification using Nickel-Iminodiacetic acid resin, with a wash buffer containing 20 mM tris, 100 mM NaCl, 20 mM imidazole, and 0.2% (w/v) n-Dodecylphosphocholine (DPC), at pH 8.0, followed by elution buffer (20 mM tris, 100 mM NaCl, 300 mM imidazole, 0.2% DPC, at pH 8.0). Approximately 35 to 40 mg of purified *Bj*SemiSWEET were obtained from 1 liter of M9 culture.

### Preparation of *Bj*SemiSWEET cellular membrane samples

*Bj*SemiSWEET cellular membrane samples were derived from *E. coli* cells. The total cellular membrane fraction was obtained through density gradient centrifugation and subsequently purified using an enhanced sucrose gradient centrifugation method (*26*). The cellular membranes were resuspended in lysis buffer (20 mM tris, 100 mM NaCl, pH 8.2) and homogenized via gentle sonication before centrifugation. A layering technique was then applied, sequentially adding 2.7 ml of 60% (w/v) sucrose, 3.5 ml of 51% (w/v) sucrose, 3 ml of 35% (w/v) sucrose, and 4 ml of the total membrane suspension from bottom to top in a tube. Centrifugation was carried out using an SW-40Ti rotor (Beckman) at 169,818*g* for 16 hours. The inner membrane fraction between the 35% and 51% (w/v) sucrose layers was collected, as well as the outer membranes at the 51% to 60% sucrose interface. To collect the inner membranes, 2 ml of the sample was diluted with water to fill an 8.9-ml Beckman centrifuge tube and centrifuged at 419,832*g* for 3 hours. After freeze-drying, rehydration with 30% (w/w) water was followed by packing into a 3.2-mm thin-walled rotor for ssNMR experiments.

### Preparation of *Bj*SemiSWEET liposome samples

We prepared *Bj*SemiSWEET protein liposomes following a dialysis method. Purified *Bj*SemiSWEET was combined with DMPC:DMPG = 4:1 lipid at a ratio of 1.25 (w/w). Lipid stocks at a concentration of 5 mg/ml were created through hydration and ultrasonication, and subsequently dissolved in 2% (w/v) *n*-octyl-β-glucoside (OG). These lipids were then mixed with the purified *Bj*SemiSWEET, leading to a dilution of the protein concentration to 0.5 mg/ml using elution buffer. After a 2-hour incubation at 18°C, the mixture underwent dialysis for 7 days at 12°C against an external dialysis buffer comprising 20 mM tris at pH 7.7, supplemented with 0.1 mM dithiothreitol (DTT). The dialysis buffer transitioned to 10 mM tris on the sixth day and to 5 mM tris on the seventh day. *Bj*SemiSWEET protein liposomes were harvested via ultracentrifugation at 419,832*g* for 3 hours and subsequently freeze-dried. The desiccated complexes were reconstituted with 30% (w/w) water and loaded into a 4-mm thin-walled rotor for ssNMR experiments.

### Sucrose transport activity of *Bj*SemiSWEET

Sucrose transport experiments were assayed using established protocols (*38*). The purified *Bj*SemiSWEET protein was reconstituted into liposomes as described above. Protein-free liposomes were prepared similarly, except that the protein solution was replaced with the buffer used for the final purification step. After dialysis to remove the detergent, these liposomes were subjected to five freeze-thaw cycles with supra-sufficient sucrose FRET sensor FLIPsuc90mΔ1V, followed by extrusion through a 400-nm polycarbonate (PC) membrane. Unincorporated FLIPsuc proteins were removed via two rounds of ultracentrifugation. The resulting proteoliposomes encapsulating FLIPsuc were resuspended in dialysis buffer (20 mM tris, pH 7.7). Fluorescence intensity at the peak emission wavelength (524 nm) was then monitored. Functional liposomes exhibit a gradual decrease in fluorescence upon sucrose binding. After the fluorescence intensity stabilized for approximately 100 s, sucrose was added to a final concentration of 20 mM, and the changes in fluorescence intensity were subsequently recorded.

### Quantitative characterization of native cellular membranes components

Methods for measuring protein components in different samples are provided in previous studies (*26*, *37*).

#### Quantitative characterization of total proteins

Samples were prepared identically to *Bj*SemiSWEET cellular membranes, except M9 medium was fully isotope-labeled (^15^N) to tag all proteins. Total protein content was determined by comparing the integrated ^15^N NMR signal intensity of native membranes to *Bj*SemiSWEET proteoliposome standards.

#### Quantitative characterization of labeled proteins

The procedures for sample preparation were identical to those described in the *Bj*SemiSWEET cellular membrane sample preparation. The amount of labeled proteins was calculated by analyzing the ^15^N signal intensity as reference to that of *Bj*SemiSWEET in synthetic lipid bilayers, approximately. The content of unlabeled background proteins could be calculated by subtracting the labeled protein content from the total protein content.

#### Quantitative characterization of labeled BjSemiSWEET

The procedures for sample preparation were identical to those described in the *Bj*SemiSWEET cellular membrane sample preparation. 2D NCA NMR distinguished *Bj*SemiSWEET signals from background. Cross-peak intensities were normalized to synthetic liposome controls. The content of labeled background proteins could be calculated by subtracting the labeled *Bj*SemiSWEET content from the label protein content. All results are shown in table S1.

### ssNMR experiment settings

ssNMR experiments, including NCACX, NCOCX, CONCA, and NCACB, were conducted on *Bj*SemiSWEET’s cellular membrane samples using a standard Bruker Advance 800 MHz spectrometer equipped with a 3.2-mm E-free HCN MAS probe spinning at 10.5 kHz and 273 K. In these experiments, selective polarization transfer between ^13^C and ^15^N was achieved with SPECIFIC CP (*57*), and ^13^C-^13^C correlations were established through dipolar-assisted rotational resonance (DARR) recoupling (*58*). Distance constraints for ^13^C-^13^C correlations were determined through 2D CORD experiments (*42*) on ^15^N,2-^13^C and ^15^N,1,3-^13^C glycerol-labeled *Bj*SemiSWEET samples. 2D CORD spectra were acquired with mixing times of 100 and 500 ms. Chemical shifts for ^13^C were referenced to adamantane (40.48 ppm methylene carbon), while ^15^N shifts were indirectly referenced to liquid NH_3_ based on the gyromagnetic ratios. For investigating the conformational exchange of *Bj*SemiSWEET in cellular membranes, 2D ^13^C-^13^C and ^13^C-^15^N correlation spectra were recorded at various temperatures. The mixing time for the 2D ^13^C-^13^C CORD experiments was 50 ms, with experimental temperatures set at −10°C, 0°C, and 12°C. In the 2D ^13^C-^15^N NCA experiments, the Double Cross-Polarization mixing time was 5 ms, and the experimental temperatures ranged from 0°C to 17°C. 2D CEST-NCA experiments were used to selectively monitor ^15^N nuclei during exchange. ^15^N magnetization was generated via cross-polarization (CP), followed by chemical shift encoding in the t1 dimension. Two 90° pulses aligned the ^15^N magnetization along the *z* axis for chemical exchange. During the exchange period, a weak saturation field (B1 = 0 to 16 Hz in this study) was applied at a specific frequency for a duration Tsat. Subsequently, CP transferred the ^15^N magnetization to ^13^C for chemical shift encoding in the t2 dimension. The CEST irradiation frequency was centered on conformation 1 (120.5 ppm). Saturation power was incrementally increased (0 Hz → 12.5 Hz → 16 Hz; Tsat = 500 ms), and three NCA spectra were recorded. Signal intensities were normalized to the B1 = 0 Hz reference spectrum. All isolated peaks were analyzed for intensity variations under different B1 fields. 2D CSA-CODEX-NCA experiments use two rotor-synchronized 180°composite pulse blocks. The first block recouples ^15^N anisotropic chemical shifts, inducing signal attenuation, while the second (phase-inverted) block restores the signal. Two separated 90° pulses between the composite blocks generate a stimulated spin echo. A mixing period (tm) is inserted between the 90° pulses. Contributions from spin diffusion (T1 relaxation) during tm are minimized by introducing a fixed delay (tz) after the second CSA recoupling block. The total time (tm + tz) remains constant, ensuring identical T1 effects in both. Normalized exchange signal (*S*/*S*_0_) reflects pure chemical exchange, and fitting *S*/*S*_0_ versus tm yields slow exchange parameters. NCA spectra were recorded with mixing times tm = 0.2, 3, 5, 10, 15 ms. NCA and DARR experiments on *Bj*SemiSWEET’s synthetic membrane samples, namely, DMPC/DMPG and POPC/POPG, were conducted on a wide-bore Varian VNMRS NMR spectrometer operating at 600 MHz (14.1 T) using a 4-mm triple-resonance T3-HXY MAS probe with a MAS speed of 8 kHz. Typical 90° pulse lengths for MAS NMR experiments were 3.8 μs (^1^H), 4.3 μs (^13^C), and 6.1 μs (^15^N), with a proton ^1^H radio frequency (RF) field of approximately 65 kHz used for acquisition and decoupling during indirect evolution. Data processing was conducted using NMRpipe (*59*), and analysis was performed in Sparky.

### Structural restraints

#### Dihedral angle restraints

Dihedral angles were determined based on N, C′ Cα, and Cβ chemical shifts using TALOS+ (fig. S1C) (*46*). Only angles classified as “good” by TALOS+ were used as dihedral angle restraints, with associated errors provided by the program. This selection process led to 79 phi/psi restraints.

#### Hydrogen-bond restraints

The intra-helical hydrogen-bond restraints were implemented when both the chemical shift index (*60*) and TALOS+ suggested helical conformations for the involved residues. This intra-helical hydrogen-bond restraint was characterized by the distance between the donor oxygen O_i_ and the acceptor nitrogen N_i+4_, set within 1.5 to 3.5 Å. A total of 74 hydrogen bonds were identified for conformation I, and 75 for conformation II.

#### ^13^C-^13^C distance restraints

^13^C-^13^C distance restraints were deduced from two ^13^C-^13^C CORD spectra with a 500-ms mixing time for 2-*Bj*SemiSWEET and 1,3-*Bj*SemiSWEET, PAR spectra with a 20-ms mixing time, and CHHC spectra with a 500-μs mixing time of uniformly ^13^C, ^15^N–labeled *Bj*SemiSWEET (table S2). Peaks corresponding to these distance constraints were identified following a structured process: (i) Cross peaks were manually selected in Sparky based on criteria such as symmetry, resolution, and a signal-to-noise ratio (SNR) of ≥6 in CORD spectra. (ii) Assignments of the cross peaks were made, with a tolerance of ~0.2 ppm set for both w1- and w2-dimensional ^13^C chemical shifts, considering the linewidth of the peaks (0.2 to 0.6 ppm) in the CORD spectra. This procedure produced some ambiguous assignments. (iii) To improve assignment accuracy, ambiguity was resolved using the strategies outlined in fig. S6. Initially, spectral peaks were classified based on intra- or inter-monomer correlations. Ambiguity in intramonomeric correlations can be notably reduced by leveraging the CS-Rosetta dimer structure of *Bj*SemiSWEET. Assignments exceeding the 9.0-Å limit between correlated atoms were omitted. Because of template uncertainties, a violation tolerance of 2.0 Å was set. For instance, the assignment of peak (67.1, 46.3) ppm was resolved by evaluating all possible assignments on the CS-Rosetta structure, with only the P63Cɑ-G10Cɑ intramonomer assignment meeting the criteria (fig. S6C). Ambiguity in intermonomeric correlations was reduced by referencing the CS-Rosetta dimer structure of *Bj*SemiSWEET. Correlated atoms beyond a 9.0-Å distance in the dimer structure were not considered for assignment. Additionally, a 2.0-Å violation tolerance was implemented due to template uncertainties. For example, the assignment of peak (62.1, 46.3) ppm was clarified by examining all potential assignments on the lowest energy CS-Rosetta structure, with only the Y55Cɑ-G10Cɑ intermonomer assignment meeting the criteria (fig. S6D).

### Dimer structures of *Bj*SemiSWEET predicted by CS-Rosetta

The initial dimer structure of *Bj*SemiSWEET was predicted using the standard POMONA/CS-RosettaCM protocol from the CS-Rosetta package (*61*, *62*). A total of 82 backbone ^15^N, ^13^C_α_, ^13^C′, and 75 ^13^C_β_ chemical shifts, along with 16 initial ambiguous distance restraints (table S4 and fig. S5, A and B), were used in the fragment and template selection process. During the subsequent structure generation phase, only fragments and templates of Protein Data Bank (PDB)proteins having under 30% sequence similarity to *Bj*SemiSWEET proteins were considered. In the process of generating structures, we have excluded nine homologous PDB protein structures (5UHQ, 4X5N, 4X5M, 4QNC, 4QND, 5UHS, 4RNG, 5CTH, and 5CTG). It is worth noting that the calculations were conducted in membrane environments. Ten thousand all-atom models were created, and the lowest energy models meeting with distance restraints were chosen as the initial dimer structures of *Bj*SemiSWEET for further analysis. For conformation I, six structures with a backbone atom RMSD of 2.1 ± 0.6 Å were chosen, and for conformation II, nine structures with a backbone atom RMSD of 3.5 ± 0.7 Å were selected (fig. S6, A and B).

### Assignments of intramonomer distance constraints

In the 500-ms CORD spectra of 2- and 1,3-*Bj*SemiSWEET, only isolated peaks with diagonal symmetry, appropriate linewidths (0.2 to 0.6 ppm), and a satisfactory SNR (≥6) were considered for the assignments with a chemical shift tolerance of 0.2 ppm. To minimize ambiguity in assigning intramonomer distance constraints, the CS-Rosetta dimer structures of *Bj*SemiSWEET were used. At this stage, the potential assignments of the intermonomer were temporarily set aside. Assignments involving intramonomer distances between correlated atoms exceeding a maximum limit of 9.0 Å in the dimer structure were omitted. A total of 30 long-range intramonomer constraints were acquired for conformation I, and 29 long-range intramonomer constraints were obtained for conformation II.

### Assignments of intermonomer distance constraints

The initial CS-Rosetta dimer structures of *Bj*SemiSWEET were used to identify intermonomer contacts in the 500-ms CORD spectra. Peaks in the CORD spectra indicating intermonomer correlations were assigned based on specific criteria: (i) The tolerance window of chemical shift was defined as half-line width; (ii) only peaks with diagonal symmetry, appropriate linewidths (0.2 to 0.6 ppm), and adequate SNR (≥6) in CORD spectra were assigned; (iii) the upper distance limit for the assigned constraints was set to 9.0 Å with a violation tolerance of 2.0 Å due to uncertainty in the initial template. In total, 26 intermonomer constraints were acquired for conformation I, and 36 intermonomer constraints were obtained for conformation II.

### Determination of *Bj*SemiSWEET dimer structures using Xplor-NIH

Dimer structures of *Bj*SemiSWEET were de novo calculated using the Xplor-NIH program (version 2.47) (*48*) with a set of distance restraints. Standard terms for bond lengths, bond angles, and improper angles were used in the calculations to maintain accurate covalent geometry. Statistical torsion angle potentials, gyration volume terms, and the hydrogen-bond database term HBPot were used to enhance the structural accuracy, particularly in hydrogen-bond geometry. The folding calculations for the full-length single-stranded *Bj*SemiSWEET were extrapolated from first-order sequence extensions of the chain. The two monomers were constrained to maintain identical structures during the annealing process through the noncrystallographic symmetry term PosDiffPot and the translational symmetry term DistSymmPot.

A total of 1000 structures were de novo generated through MD-simulated annealing in torsion angle space with two consecutive annealing schedules, followed by final gradient minimization in Cartesian space. Each structure was subject to specific constraints: 430*2 unique nonredundant ^13^C-^13^C distance constraints, 74*2 hydrogen-bond constraints, and 79*2 TALOS-N–derived torsion angle constrained chains for conformation I, and 419*2 unique nonredundant ^13^C-^13^C distance constraints, 75*2 hydrogen-bond constraints, and 79*2 TALOS-N–derived torsion angle constrained chains for conformation II, per *Bj*SemiSWEET dimer structure.

The structural calculations commenced with constant temperature MD at 3500 K, running for a duration of the shorter of 800 ps or 10,000 steps, with the time step allowed to vary to uphold constant energy within acceptable limits. The temperature was sequentially decreased by 25 K. At each temperature, the kinetics were executed for the duration of 2 ps or 500 steps. The force constant for the distance constraint was increased from 1 to 300 kcal mol^−1^ Å^−2^. The dihedral angle constraint was deactivated during high-temperature kinetics at 3500 K but was reinstated during simulated annealing with a force constant of 300 kcal mol^−1^ rad^−2^. The slew volume force constant was extended from 0.002 to 1. Subsequently, the structure was minimized using Powell’s energy minimization scheme, resulting in 1000 structures. The first 10 lowest energy structures exhibited RMSD values of 1.5 ± 0.2 Å for conformation I and 2.5 ± 0.3 Å for conformation II at backbone atoms. Structural quality was assessed using PROCHECK (*63*) and MolProbity (*64*), and detailed statistics were provided in table S6.

### MD simulations of *Bj*SemiSWEET conformational distribution in cellular membranes and lipid bilayers

To explore the conformational energy variances of *Bj*SemiSWEET across different membrane environments, accelerated sampling techniques via metadynamics were used. First, membrane systems with different environments were constructed, including artificial membrane systems and *E. coli* inner membrane systems. The artificial membrane system primarily featured DMPC and DMPG lipids in a ratio of DMPC:DMPG = 80:20, while the *E. coli* inner membrane composition included 1-palmitoyl-2-oleoyl-sn-glycero-3-phosphoethanolamine (POPE), 1-palmitoyl-2-oleoyl-sn-glycero-3-phosphoglycerol (POPG), and cardiolipin (CDL) lipids in a ratio of PE:PG:CDL = 75:13:12. Reaction coordinates were defined by the distances between gating residues at the outer and inner gates: Reaction coordinate 1 (CV1) is the distance between residue D61 Cα atoms on the two monomers, and reaction coordinate 2 (CV2) is the distance between residue D36 Cα atoms on the two monomers. Throughout the simulations, all replicas were subjected to bias potentials every 2500 steps, with a height of 0.5 kJ/mol, a width of 1.0 rad, and a bias factor of 32. Conformations, collective variable (CV) values, and energies were saved during the simulations. Conformations were exchanged between neighboring replicas every 1000 steps, with a simulation duration time of 1.0 μs.

### MD simulations of sucrose transport through cellular membranes by *Bj*SemiSWEET

To acquire detailed thermodynamic insights into ligand dissociation, we used FM simulations to compute the free energy landscape of sucrose translocation via *Bj*SemiSWEET. To study the complete process of sucrose transport, we divided it into two steps: the binding process and the release process. During the binding process, constraints were applied near the intracellular gate of *Bj*SemiSWEET to inhibit sucrose release. In the release process, constraints were positioned near the extracellular gate of *Bj*SemiSWEET to block sucrose from traversing the outer gate. The concept of FM sampling entails establishing a funnel-shaped rigid constraint, where the conical region covers the binding site, and the cylindrical region extends into the solvent area beyond the binding site.

The funnel constraint in this study is characterized by several parameters: Zcc defines the length of the conical region, α defines the opening angle of the conical region, and Rcyl defines the radius of the cylindrical region. Here, Zcc is set to be 1.2 nm, α = 0.75 and Rcyl is set to be 0.2 nm for the enter process, and Zcc = 1.3 nm, α = 0.9, Rcyl = 0.2 nm for the release process. By using these parameters, the ligand is confined within the funnel region, which reduces the computational cost of sampling. The entry and release processes are delineated by two reaction coordinates. In the entry process, the reaction coordinates comprise the distance between residue D61 Cɑ atoms near the outer gate and the ligand’s *z*-direction position (perpendicular to the membrane surface). In the release process, the reaction coordinates include the distance between residue D36 Cɑ atoms near the inner gate and the ligand’s *z*-direction position. During FM simulations, the Gaussian height of the bias potential is fixed at 1.0 kJ/mol, with the bias potential added every 2 ps (τ_G_). The bias factor is set to 16.

## Supplementary Materials

### This PDF file includes:

Figs. S1 to S17 Tables S1 to S6 References
