# Supplementary material for: Dynamic structures of a membrane transporter in native cellular membranes
Source: Sci Adv. 2025 Nov 12;11(46):eadv4583. doi: 10.1126/sciadv.adv4583 (PMC12609076; doi:10.1126/sciadv.adv4583)
Supplement: Supplementary file 1 — Figs. S1 to S17 Tables S1 to S6 References [file sciadv.adv4583_sm.pdf]

Science Advances
AAAS

Supplementary Materials for

Dynamic structures of a membrane transporter in native cellular membranes

Huayong Xie *et al.*

Corresponding author: Mojie Duan, mjduan@wipm.ac.cn; Jun Yang, yangjun@wipm.ac.cn

Sci. Adv. **11**, eadv4583 (2025)
DOI: 10.1126/sciadv.adv4583

This PDF file includes:

Figs. S1 to S17
Tables S1 to S6
References

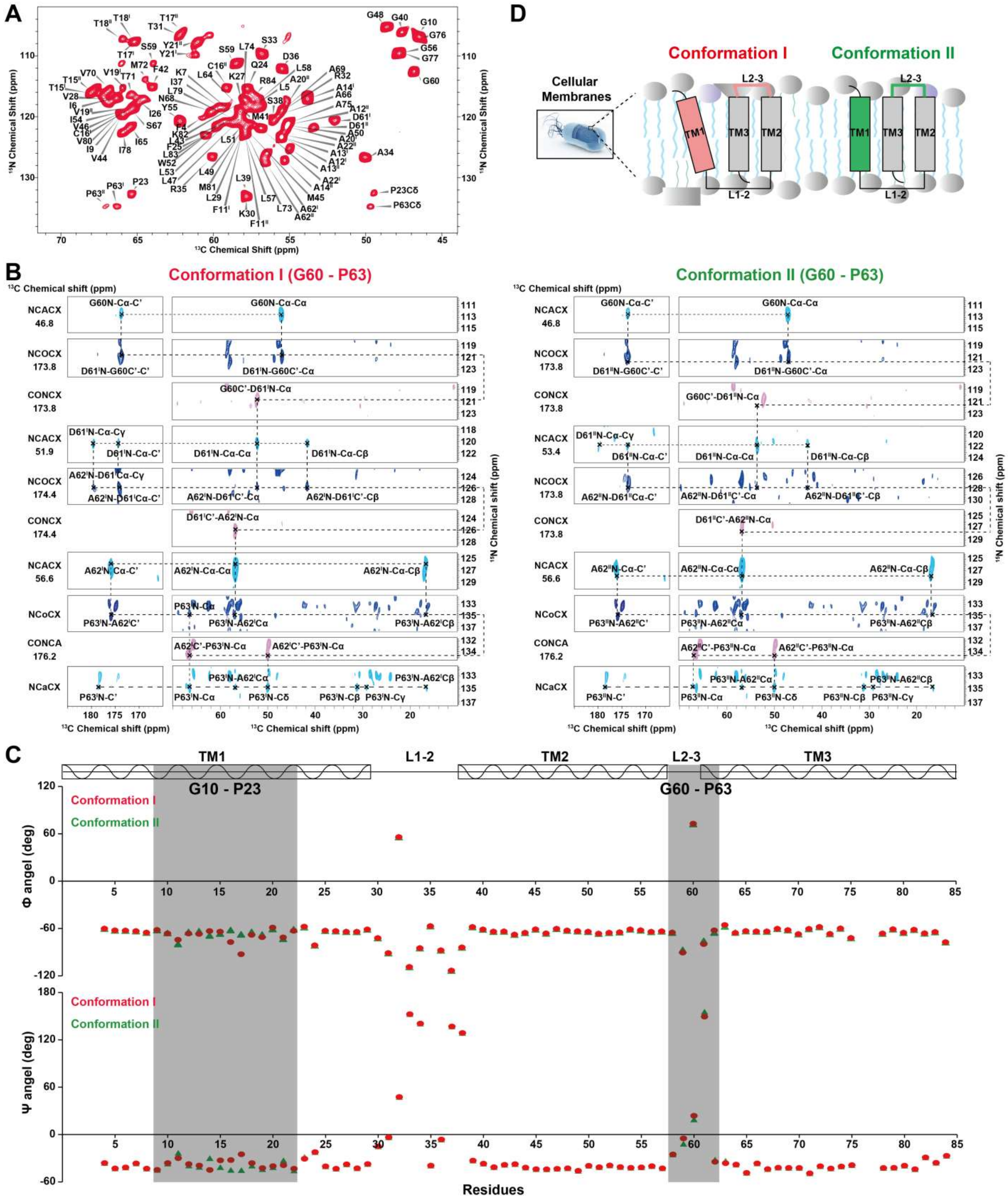

Fig. S1. Second structures of *Bj*SemiSWEET in cellular membranes. (A) Chemical shift assignments of the two BjSemiSWEET conformations in the cellular membrane labeled in the NCAspectra.These assignments have been deposited in the PDB database under IDs 9KAX and 9KBA. (B) The backbone walks of residues G60-P63 in conformation I and conformation II is traced usingthree 3D ssNMR spectra. (C) Torsion angles predicted from the chemical shifts of the twoBjSemiSWEET conformations in the cellular membranes using TALOS+ software(46). (D) Aschematic depiction of the conformation of BjSemiSWEET in cellular membranes reveals that theLoop L2-3 and TM1 regions exhibit two conformations in cellular membranes.

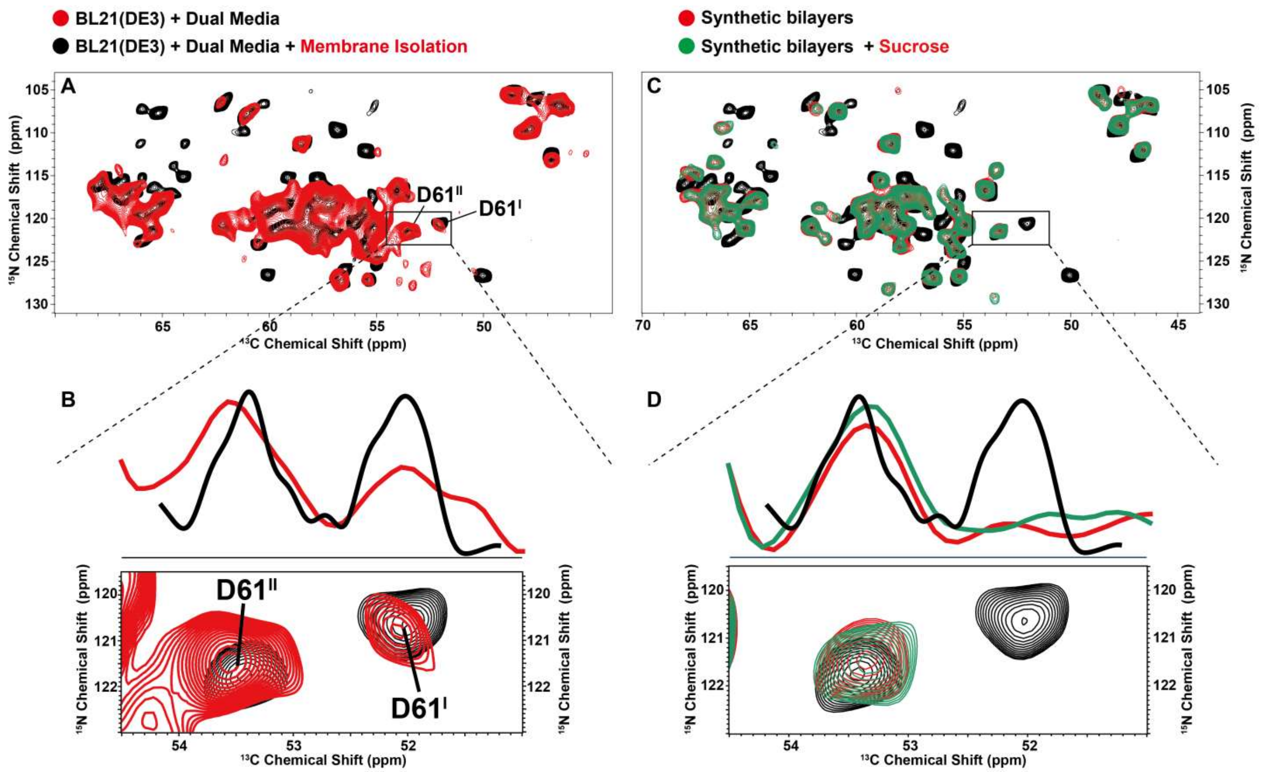

Fig. S2. Two conformational states of *Bj*SemiSWEET in native cellular membranes is intrinsic and not an artifact of residual sucrose. (A) The 2D NCA spectrum of BjSemiSWEET in a sucrose-free crude membrane preparation (red), purified without a sucrose gradient, exhibits significant spectral broadening (average linewidths: 143 Hz in ^{15}N, 151 Hz in ^{13}C). The spectrum is overlaid with that of the sucrose gradient-purified inner membrane sample (black). (B) For residues with well-resolved chemical shifts, such as D61, the impact of line broadening is negligible, allowing reliable conformational assignment. Two distinct conformational states for D61 can be clearly distinguished even in the sucrose-free crude membrane sample. (C) To further investigate whether sucrose binding artificially induces additional conformational states, excess sucrose (1:10000 molar ratio) was added to the BjSemiSWEET proteoliposome sample. The NCA spectra before (red) and after (green) sucrose addition are nearly identical. (D) Notably, no new conformational state emerged for residue D61 upon sucrose binding.

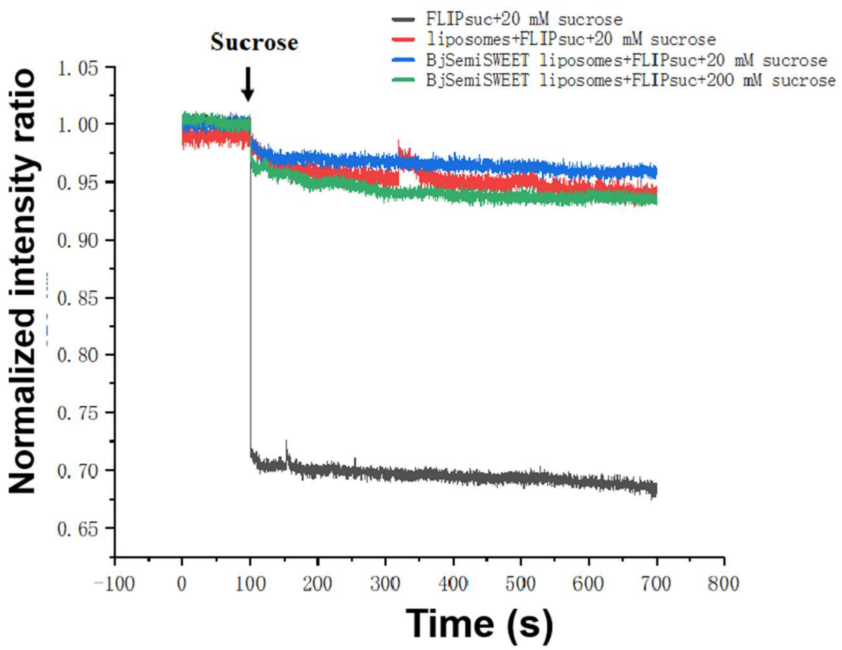

Fig. S3. Sucrose transport activity of *Bj*SemiSWEET in synthetic vesicles. While direct functional testing in native membranes is technically challenging, sucrose transport activity for *Bj*SemiSWEET in vivo has been demonstrated(*38*). Here, sucrose transport by purified *Bj*SemiSWEET reconstituted into synthetic liposomes was assayed using established protocols(*38*). Proteoliposomes and protein-free control liposomes were prepared as described in Materials and Methods. The immediate decrease in fluorescence signal upon sucrose addition confirmed functional FLIPsuc integrity (black trace; note: a minor initial signal decrease is attributable to sample dilution). *Bj*SemiSWEET-reconstituted liposomes (blue) and empty liposomes (red) exhibited nearly identical sucrose uptake rates. No significant difference in transport activity was observed, even at sucrose concentrations up to 200 mM (green).

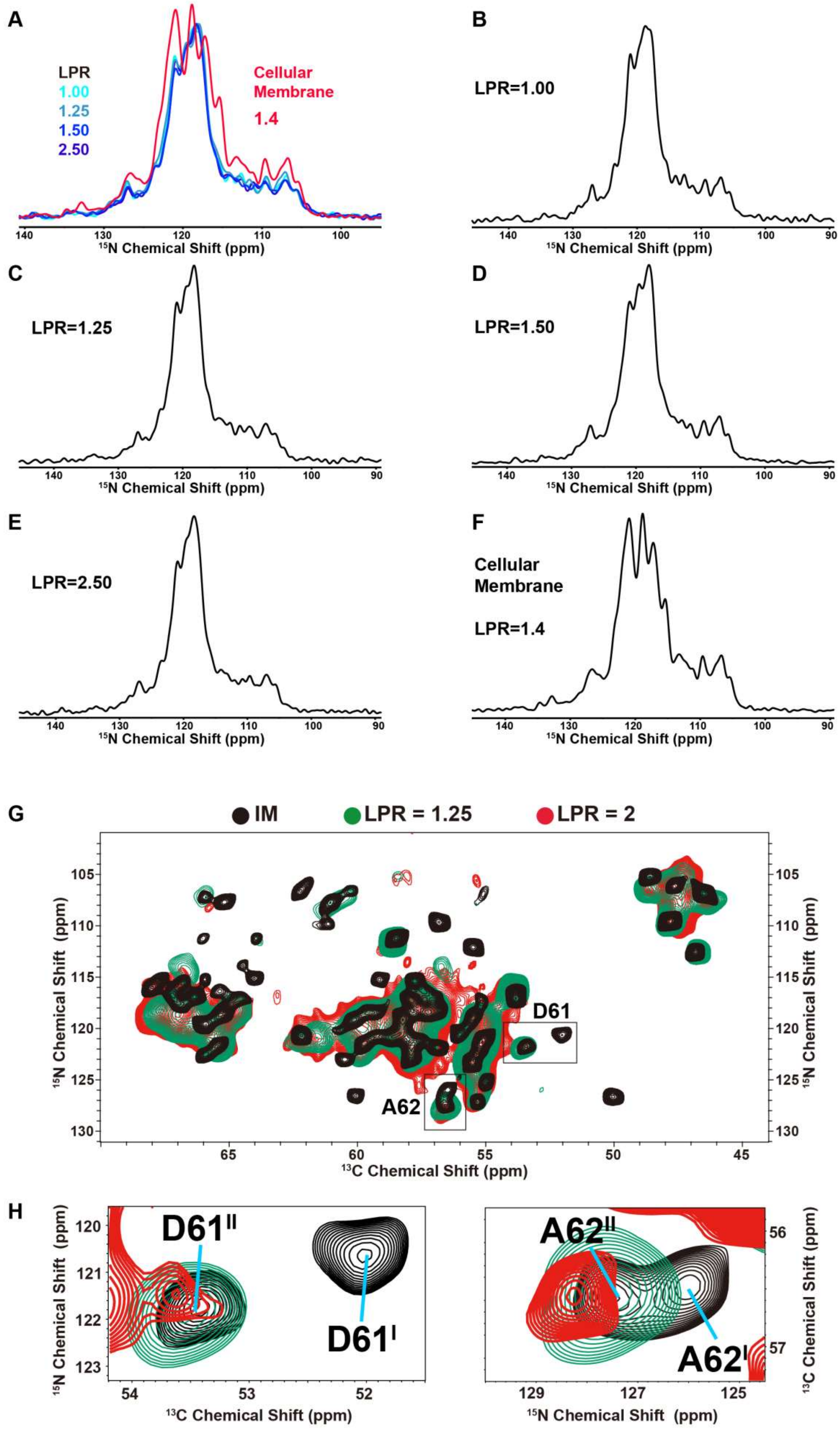

Fig. S4. BjSemiSWEET adopts a single conformation in DMPC/DMPG liposomes within the LPR range of ~1–2.5. (A–F) 1D ^{15}N spectra of BjSemiSWEET in synthetic and native membranes. (A) Overlaid ^{15}N spectra of BjSemiSWEET in synthetic membranes (DMPC/DMPG) at lipid-to-

protein mass ratios ranging from 1.0 to 2.50, compared with the spectrum in native membranes. The spectral patterns are nearly identical across different ratios in synthetic membranes but differ significantly from those in native membranes. (B–E) ^{15}N spectra of BjSemiSWEET in synthetic membranes at lipid-to-protein mass ratios of 1.0, 1.25, 1.50, and 2.50, respectively. (F) ^{15}N spectrum of BjSemiSWEET in native membranes. (G) Overlaid 2D NMR spectra of BjSemiSWEET proteoliposome samples at different LPRs. Red: DMPC/DMPG proteoliposomes at LPR = 2. Green: DMPC/DMPG proteoliposomes at LPR = 1.25. Black: Inner membrane sample (LPR ≈ 1.4). (H) Both D61 and A62 exhibit only one conformational state under LPR = 1.25 and LPR = 2 conditions.

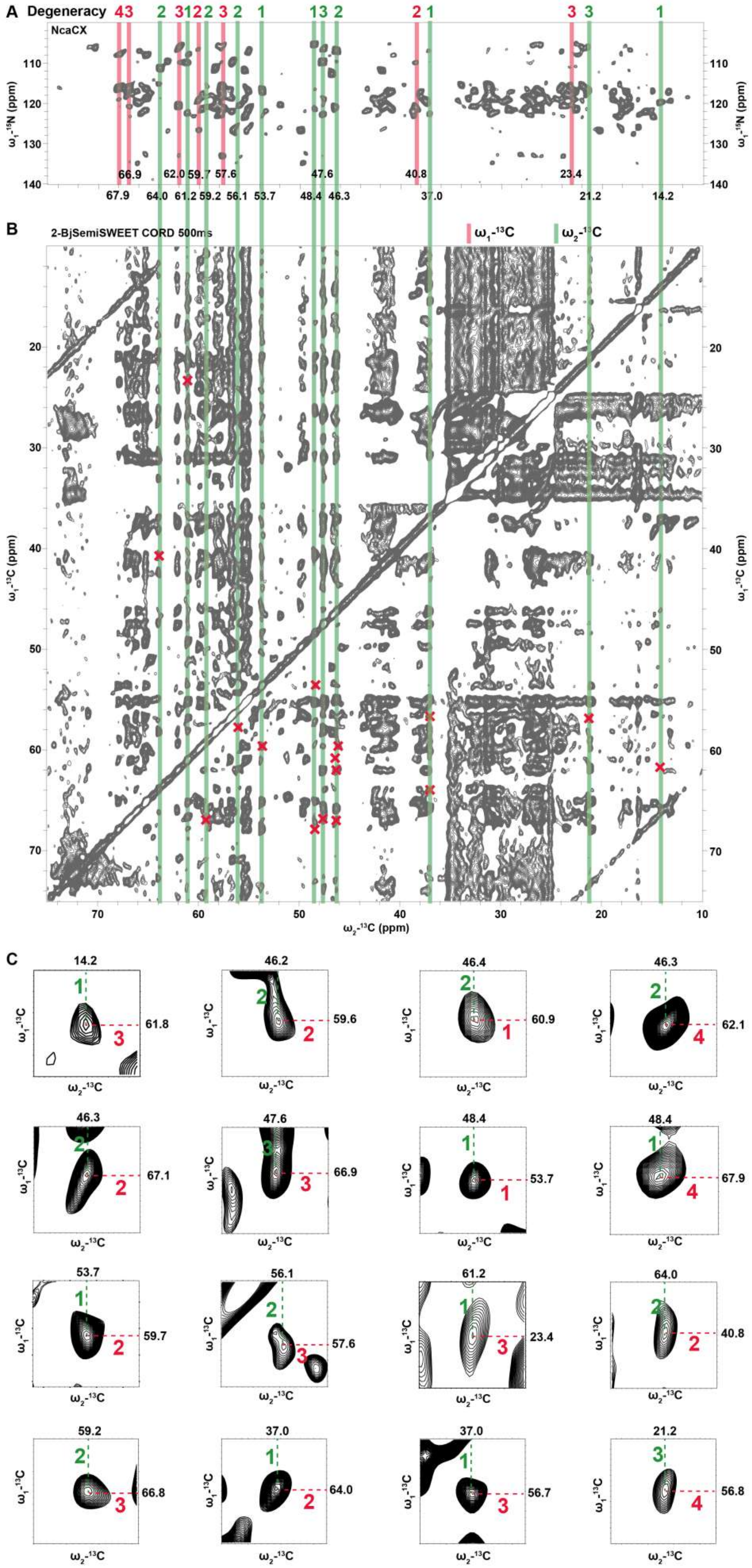

Fig. S5. Ambiguous distance restraints crucial for CS-Rosetta structure calculations. (A) The NcaCX spectrum illustrates the degeneracy of the w1-dimensional among the 16 peaks corresponding to ambiguous distance restraints with red vertical lines, and the degeneracy of the

w2-dimensional with green vertical lines. **(B)** Red crosses highlight the positions of the 16 ambiguous distance restraint peaks in the 500 ms CORD spectrum of 2-*Bj*SemiSWEET. **(C)** The cross sections of the CORD spectra show the 16 peaks corresponding to ambiguous distance restraints, verifying that all 16 peaks adhere to the criteria outlined in the methods section: only isolated peaks with diagonal symmetry, appropriate linewidths (0.2-0.6 ppm), and a satisfactory signal-to-noise ratio (SNR≥6) were considered for the assignments.

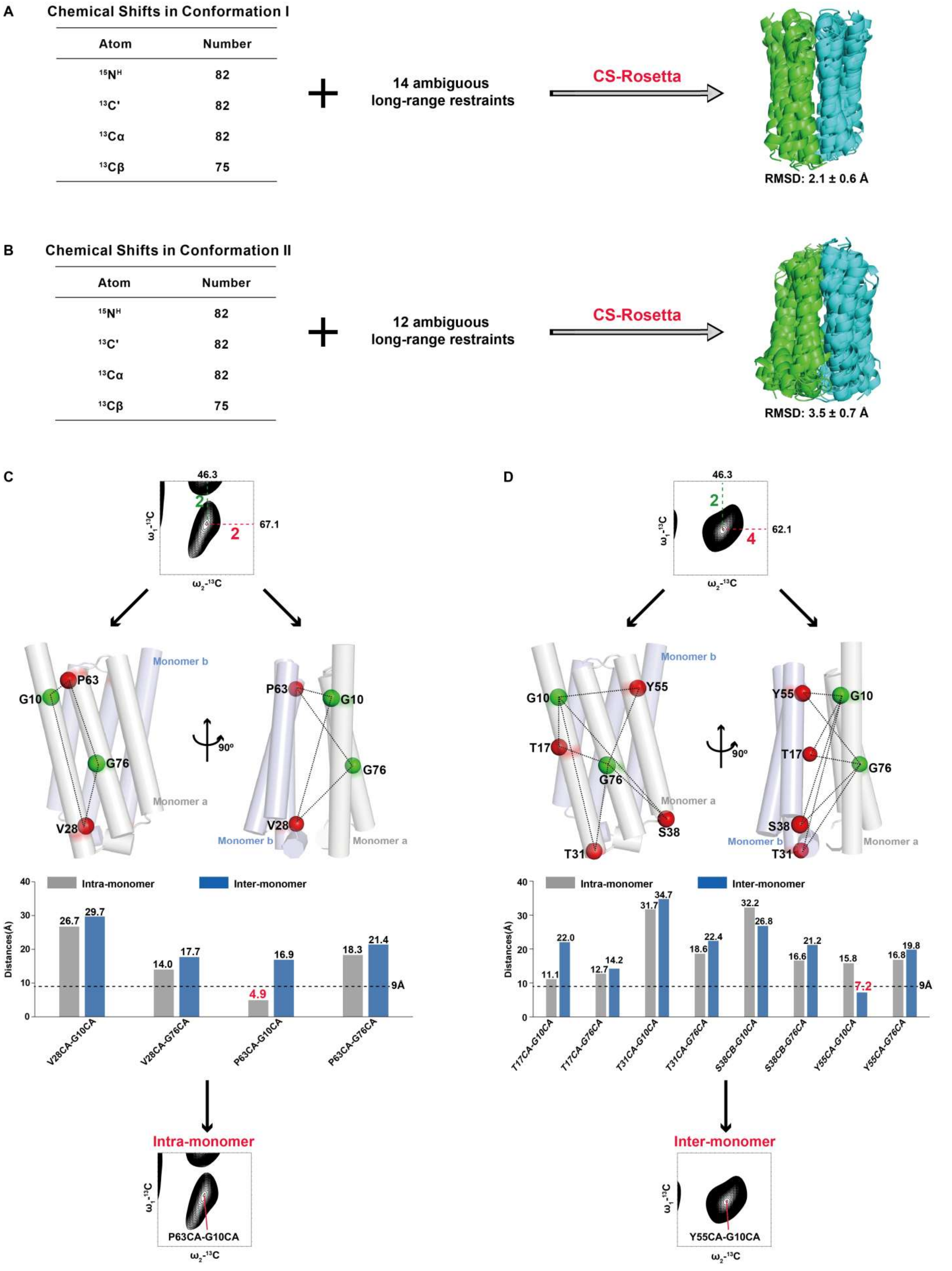

Fig. S6. Strategies for reducing ambiguity in distance constraint assignments. (A) Based on the chemical shifts of 82 backbone atoms (N, C', and Cα) and 75 sidechain atoms of residues in conformation I of *Bj*SemiSWEET, together with 14 ambiguous distance restraints (refer to **Supplementary Table 2** for details), 6 converged lowest energy structures with a backbone RMSD

of 2.1 Å were calculated by CS-Rosetta software. **(B)** Based on the chemical shifts of 82 backbone atoms (N, C', and Cα) and 75 sidechain atoms of residues in conformation II of *Bj*SemiSWEET, together with 12 ambiguous distance constraints (see **Table S3** for details), 9 converged lowest energy structures with a backbone RMSD of 3.5 Å were calculated by the CS-Rosetta software. **(C)** The strategy for assigning intra-monomeric long-range distance restraints, illustrated by the assignment of the peak at (67.1, 46.3) ppm. This peak presents two assignment possibilities in the w1 dimension for the P63Cα and V28Cα atoms, and two assignment possibilities in the w2 dimension for the G10Cα and G76Cα atoms, leading to a total of four assignment possibilities based solely on chemical shifts. The spatial positions of these potential assignments in both the w1 and w2 dimensions are illustrated using green and red markers respectively on the CS-Rosetta structure of *Bj*SemiSWEET with the lowest energy. Histograms depict the distances for all potential assignments of the (67.1, 46.3) ppm peak, where gray represents distances constrained by intra-monomeric distances and blue by inter-monomeric distances. Considering the distance threshold of 9 Å, only the assignment of intra-monomeric distance constraint P63Cα-G10Cα fulfills the criteria. **(D)** The methodology for assigning inter-monomeric long-range distance restraints, focusing on the peak at (62.1, 46.3) ppm. This peak presents four assignment possibilities in the w1 dimension for the T17Cα, T31Cα, S38Cα, and Y55Cα atoms, and two assignment possibilities in the w2 dimension for the G10Cα and G76Cα atoms. Consequently, there are a total of eight assignment possibilities based solely on chemical shifts. The spatial positions of the four potential assigning atoms in the w1 dimension are represented by green markers, and the positions of the two possible assigning atoms in the w2 dimension are indicated by red markers on the CS-Rosetta structure with the lowest energy for *Bj*SemiSWEET. Histograms display distances for all potential assignments for the peak at (62.1, 46.3) ppm, with gray denoting distances constrained by intra-monomeric distances and blue indicating distances constrained by inter-monomeric distances. Considering the distance threshold of 9 Å, only the assignment of intra-monomeric distance constraint Y55Cα-G10Cα aligns with the established criteria.

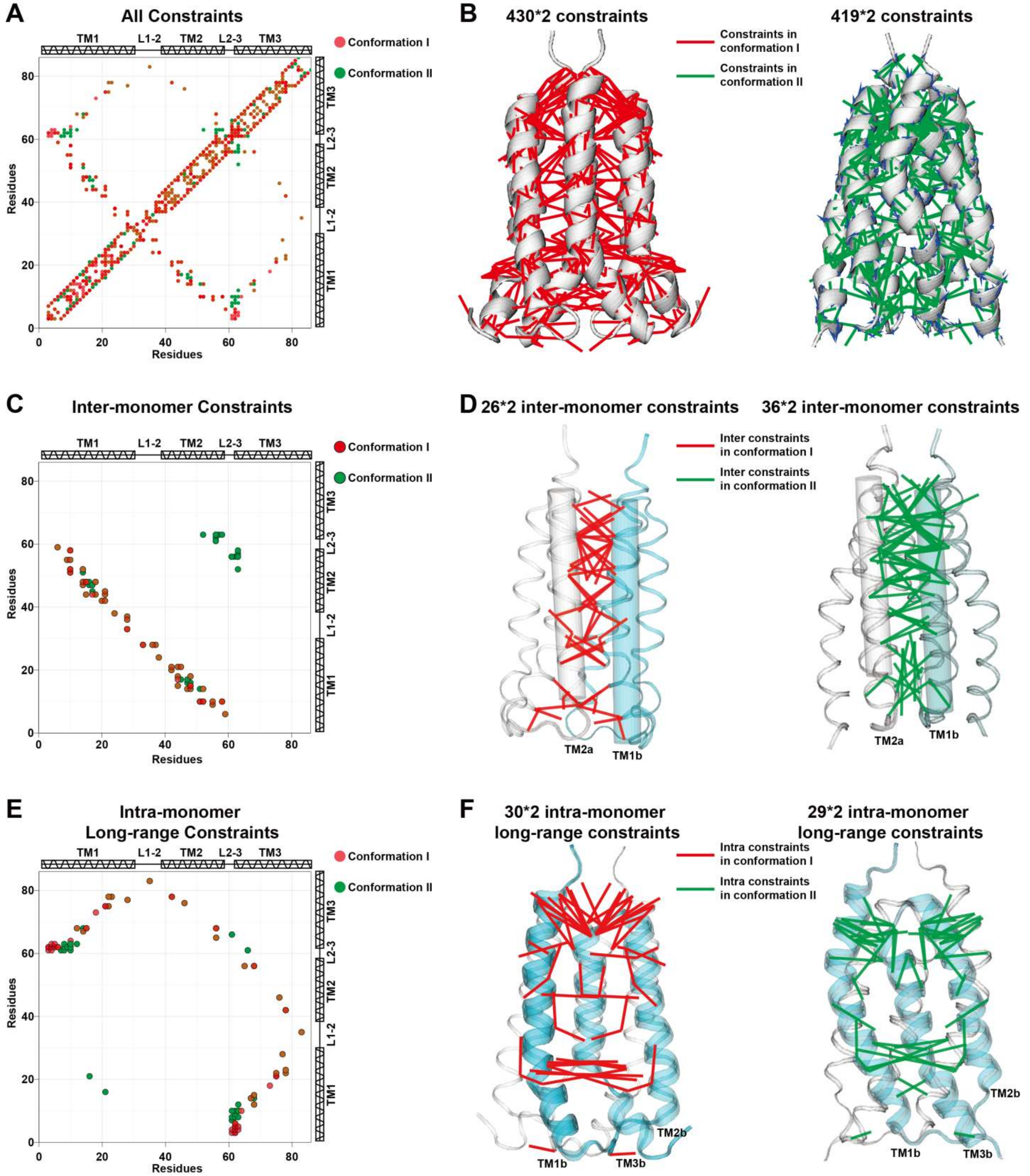

Fig. S7. Summary of distance restraints for both conformations of *Bj*SemiSWEET in the cellular membrane. (A) The contact map illustrates all distance restraints for the two conformations of *Bj*SemiSWEET, with conformation I represented by red dots and conformation II by green dots. **(B)** All 860 distance restraints (430 pairs) for conformation I are indicated by red lines on the lowest energy dimer structure of conformation I, and all 838 distance restraints (419 pairs) for conformation II are indicated by green lines on the lowest energy dimer structure of conformation II. **(C)** The contact map showcases all inter-monomeric distance restraints for both *Bj*SemiSWEET conformations, denoting conformation I with red dots and conformation 2 with green dots. Refer to **Table S4** for detailed information. **(D)** Red lines on the lowest energy dimer structure of conformation I indicate all 52 inter-monomeric distance restraints (26 pairs), whereas green lines on the lowest energy dimer structure of conformation II represent all 72 inter-monomeric

distance restraints (36 pairs) for conformation II. **(E)** The contact map displays all intra-monomeric long-range distance restraints for both *Bj*SemiSWEET conformations, with conformation I marked by red dots and conformation II by green dots. Further details are provided in **Table S3**. **(F)** Red lines on the lowest energy dimer structure of conformation I show all 60 intra-monomeric long-range distance restraints (30 pairs) for conformation I, while green lines on the lowest energy dimer structure of conformation II indicate all 58 intra-monomeric long-range distance restraints (29 pairs) for conformation II.

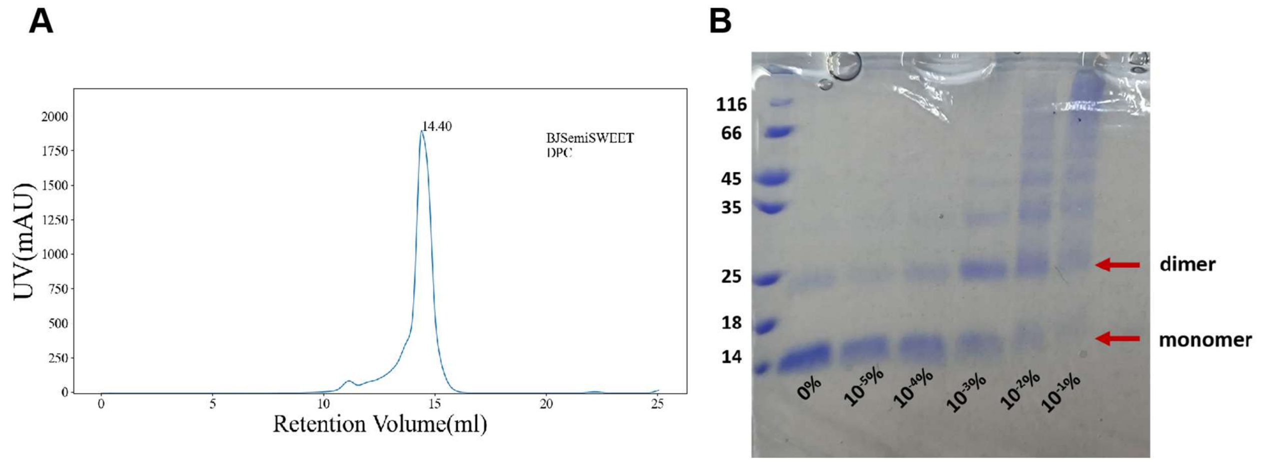

Fig. S8. Oligomerization state of *Bj*SemiSWEET in detergent and lipid vesicles. (A) Analysis of the oligomeric state of BjSemiSWEET in detergent. Gel filtration profiles of purified BjSemiSWEET on Superdex-200 (10-30 GL column) in 20 mM Tris, pH 8.0, 100 mM NaCl and 0.2% n-Dodecylphosphocholine. Purified BjSemiSWEET eluted as a monodispersed, indicating a homogeneous oligomeric state in detergent. Combined with previous cross-linking experiments demonstrating that BjSemiSWEET exists as a dimer in detergent(*40*), these results confirm that BjSemiSWEET adopts a homogeneous dimeric state in detergent. (B) Crosslinking of BjSemiSWEET in liposomes. Liposomes were prepared with a phospholipid-to-protein ratio of 50:1 (M/M) as previously described and dialyzed three times against 20 mM PB buffer (pH 7.7). Different volume concentrations of glutaraldehyde were added for crosslinking at 37°C for 30 min. The crosslinking reaction was terminated by adding 100 mM Tris. Subsequently, a native loading buffer (without DTT and SDS) was added, and the samples were analyzed by SDS-PAGE gels.The results showed that as the concentration of the glutaraldehyde increased, the content of monomers decreased while that of dimers increased, suggesting that *Bj*SemiSWEET likely exists as a dimer within the liposomes. At higher glutaraldehyde concentrations, high-molecular-weight aggregates were observed, possibly due to excessive glutaraldehyde forming polymeric networks(*65*).

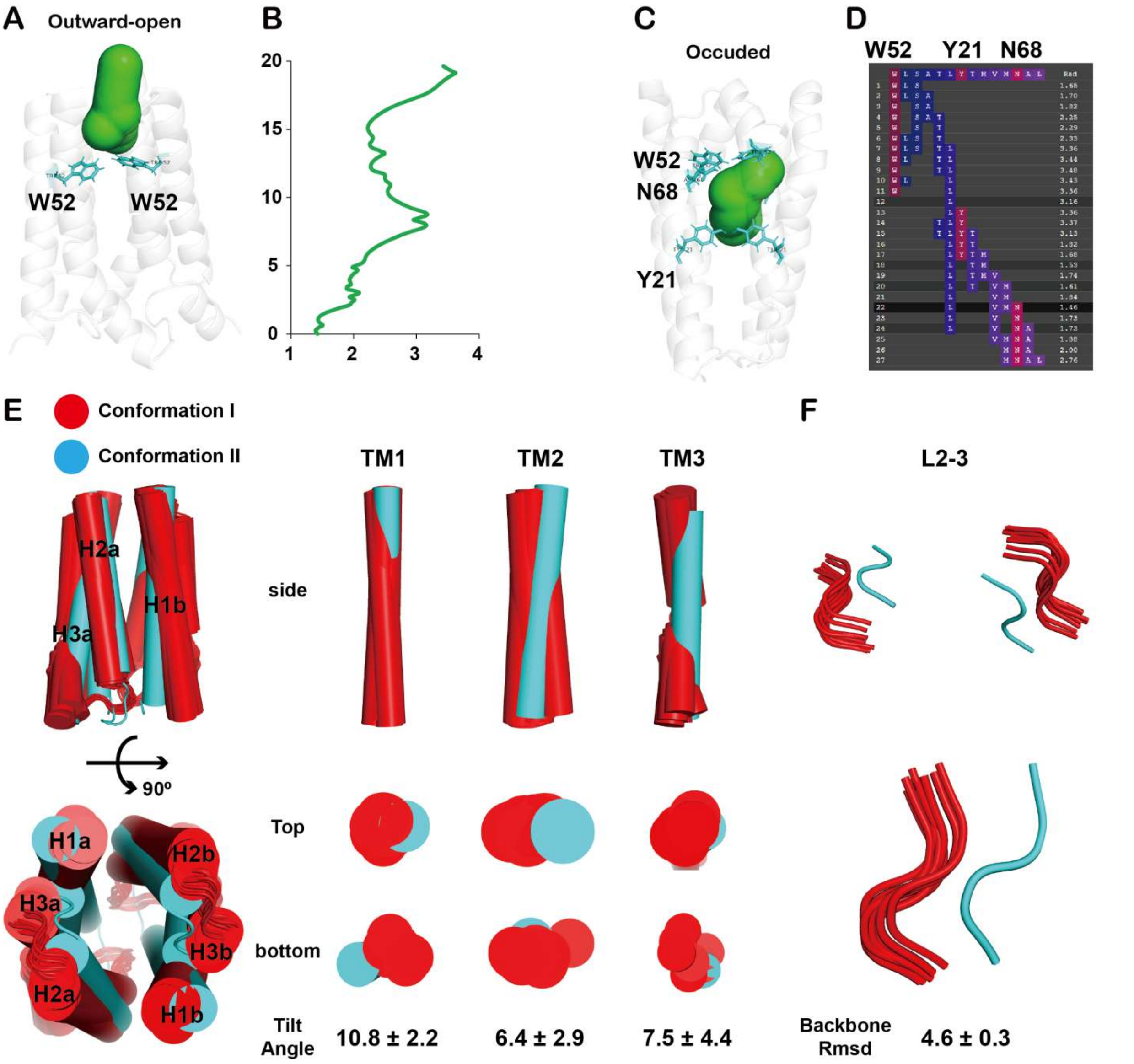

Fig. S9. Quantitative analysis of the pore size in both conformation structures of *Bj*SemiSWEET. (A-B), The pore sizes of the two *Bj*SemiSWEET conformations were quantitatively assessed using the software MOLE 2.0(*49*). Analysis reveals that *Bj*SemiSWEET conformation I exhibits an outward-open structure with an inverted conical opening extending up to 20 Å towards the extracellular space. This opening is internally bounded by residue W52, maintaining a minimum radius of 1.3 Å. **(C-D),** Conformation II features a sizable central cavity, delimited above and below by residues W52, N68, and Y21, respectively, representing an occluded conformation. These three restricted residues potentially function as substrate binding sites in other SemiSWEET proteins, suggesting the cavity's role as a substrate binding pocket. **(E-F),** The helix tilt angles of TM1 were compared, alongside an assessment of the root mean square deviation (RMSD) in Loop L2-3 between the two *Bj*SemiSWEET conformations. The superposition of the three helices from the lowest energy dimer structure of *Bj*SemiSWEET conformation II (cyan) and the ten lowest energy dimer structures of *Bj*SemiSWEET conformation I (red) was performed. The "helix tilt angle" between the two *Bj*SemiSWEET conformations was measured, using the lowest energy dimer structure of conformation II as a reference. Notably, differences exceeding 2δ were observed in the helix tilt angles of TM1 between the two conformations. The RMSD in the Loop L2-3 region between the two conformations of *Bj*SemiSWEET was approximately 5Å.

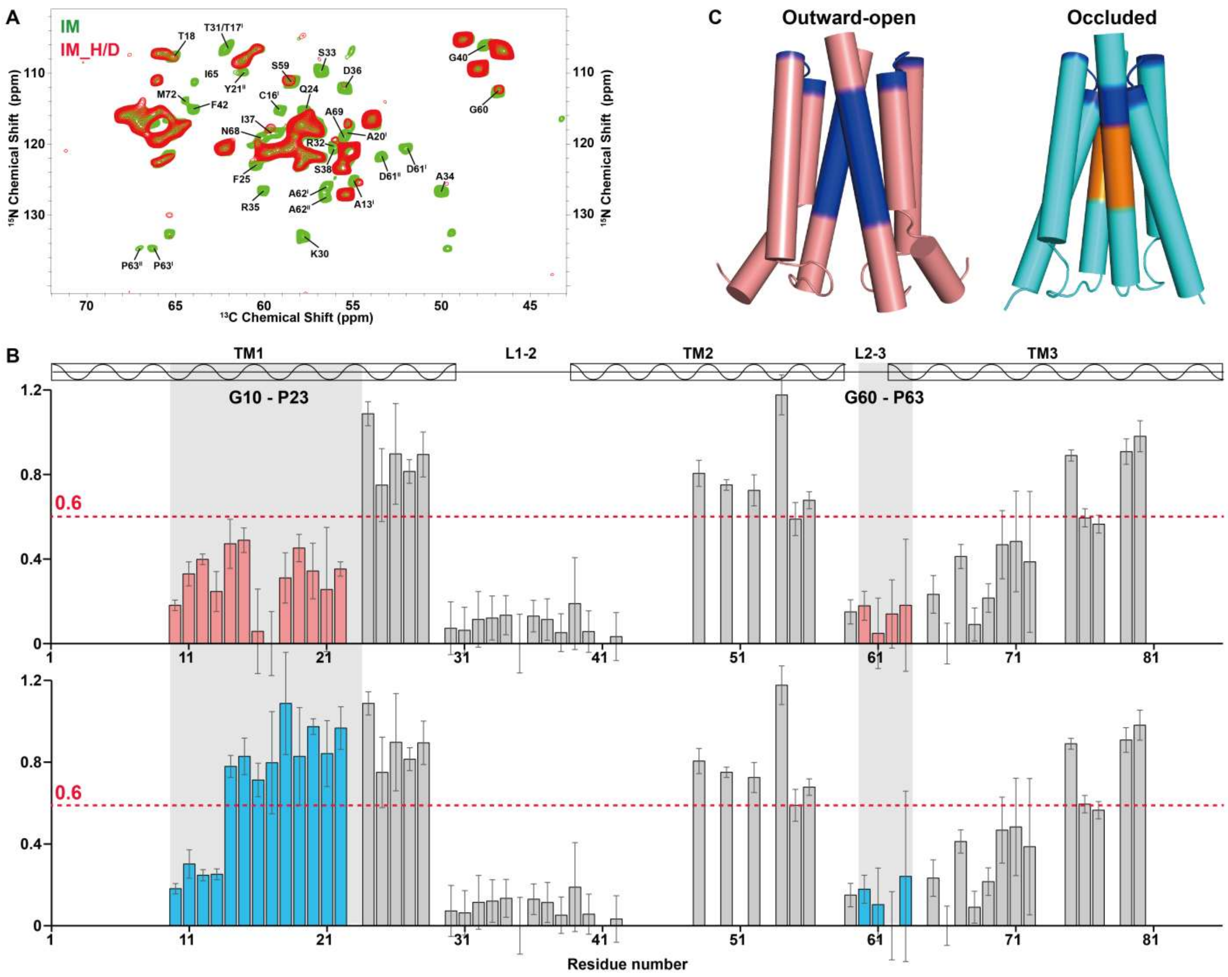

Fig. S10. Structures of the two conformations of *Bj*SemiSWEET in cellular membranes coincide with the results of H/D exchange. (A) Overlay of NCA spectra of *Bj*SemiSWEET in cellular membranes before and after H/D exchange, red and green are the NCA spectra after and before H/D exchange, respectively. **(B)** Quantitative analysis of the variations of the signal intensities in the NCA spectra before and after H/D exchange for residues of both conformations of *Bj*SemiSWEET. Residues with distinct peaks are shown in red and green for conformation I and II, respectively. Residues with a single peak are shown in gray. Errors were estimated considering the signal-to-noise ratio of the spectra and the stability of NMR spectrometer. **(C)** The signal intensity variation before and after H/D exchange of *Bj*SemiSWEET residues is visually represented on the structures of conformation I (in red) and conformation II (in cyan). A blue color denotes a signal intensity decrease of ≥50%, while orange indicates a decrease of <50%. These distinctions highlight that D_2O infiltrates between residues 14-22 in conformation I but almost not in conformation II. This alignment reinforces that conformation I exhibits an outward-open structure, whereas conformation II adopts an occluded conformation.

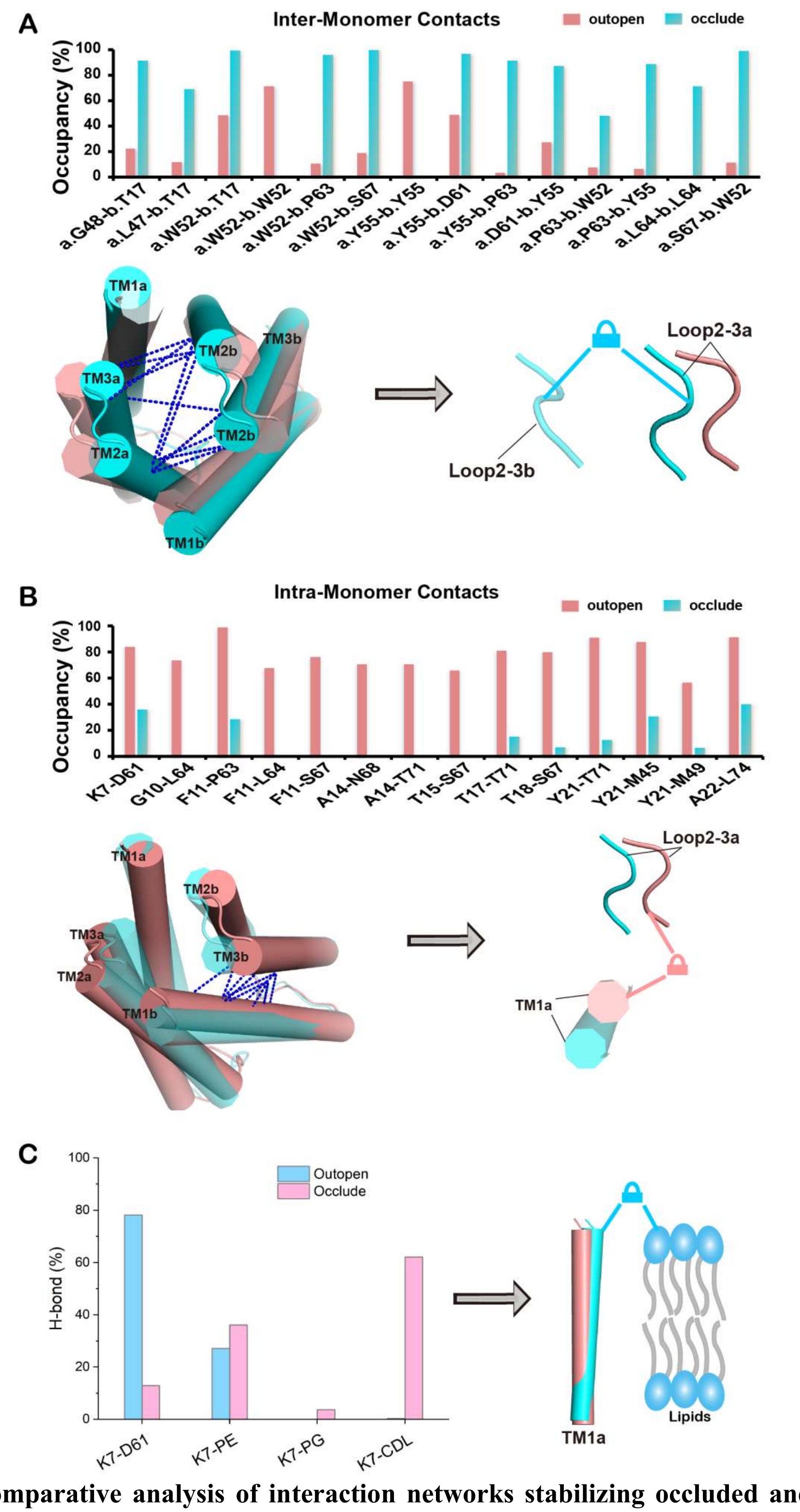

Fig. S11. Comparative analysis of interaction networks stabilizing occluded and outward-open conformations of *Bj*SemiSWEET in cellular membranes. To analyze the contacts maintaining the different conformations of *Bj*SemiSWEET, we employed all-atom molecular

dynamics simulations enhanced by advanced sampling technology to characterize the important residue-residue and residue-lipid interactions in the occluded and outward-open structures. Residue-residue interactions are characterized by residue contacts, where two residues are considered in contact when the distance between any heavy atoms in the two residues is less than 4.5 Å. Hydrogen bonds are determined based on geometric criteria between donor and acceptor atoms, the distance between the donor and acceptor atoms must be less than 3.5 Å, and the hydrogen-donor-acceptor angle must be less than 30°. **(A)** Our MD simulations highlighted a notable increase in inter-monomeric interactions within the occluded conformation, primarily driven by interactions among Loop L2-3 residues. The close proximity of Loop L2-3 leads to the sealing of the extracellular gate. Structural flexibility in the Loop L2-3 region enables smooth transitions between conformational states with low energy barriers. **(B)** Within the occluded conformation, our MD simulations indicated a decrease in intra-monomeric interactions, particularly affecting connections between residues in helices TM1 and TM3. This reduction may stem from the observed oscillatory motion in the upper segment of the TM1 helix. **(C)** Analysis of monomer-phospholipid interactions reveals an escalation in these interactions within the occluded conformation, predominantly involving specific polar residues on helix 1 (e.g., K7) and the phosphate head group of the phospholipid.

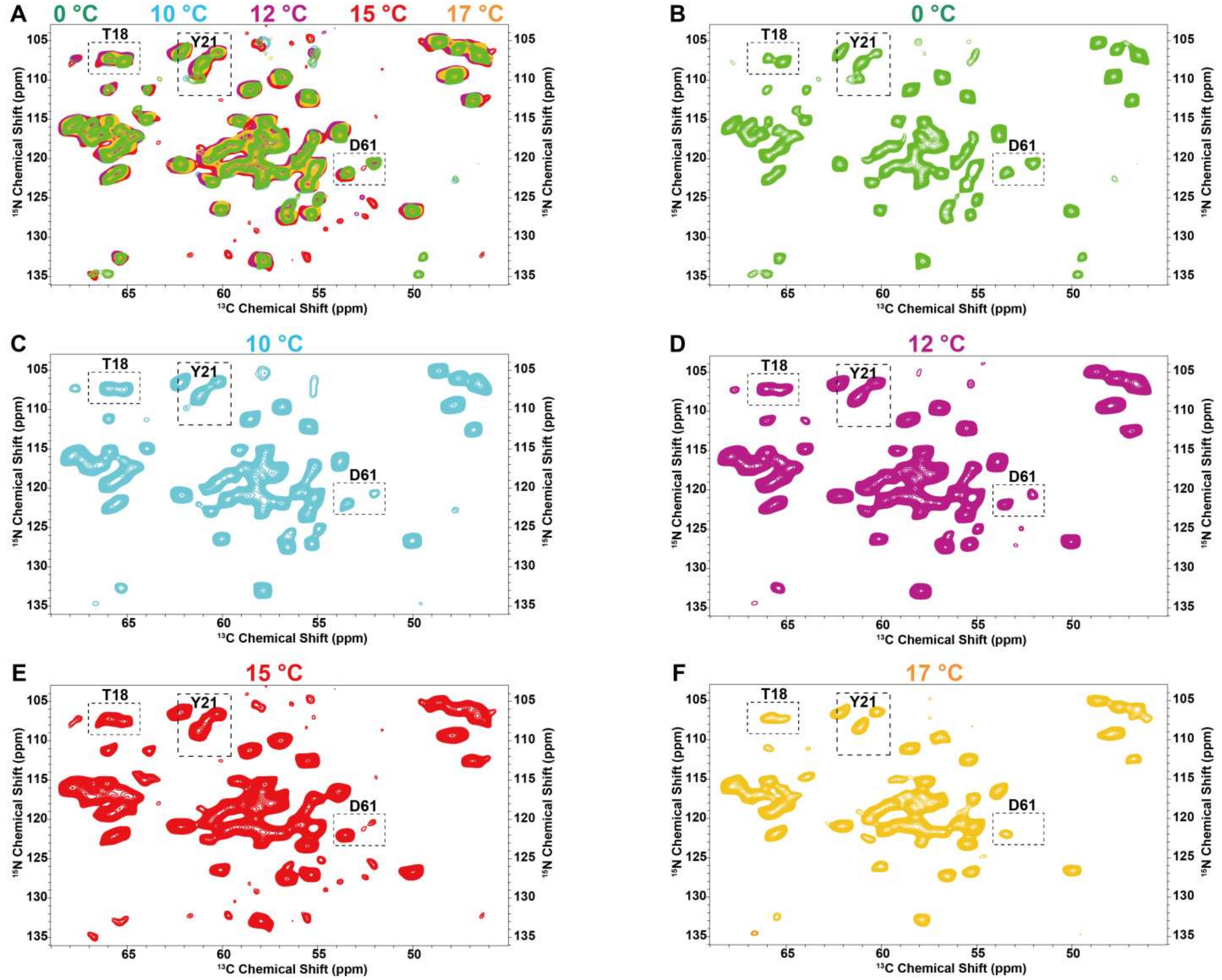

Fig. S12. **2D NCA spectra at five different temperatures.** (A) Superimposition of 2D NCA spectra at five different temperatures. (B-F) 2D NCA spectra at five different temperatures:273 K (B, green), 283 K (C, cyan), 285 K (D, purple), 288 K (E, red) and 290 K (F, orange).

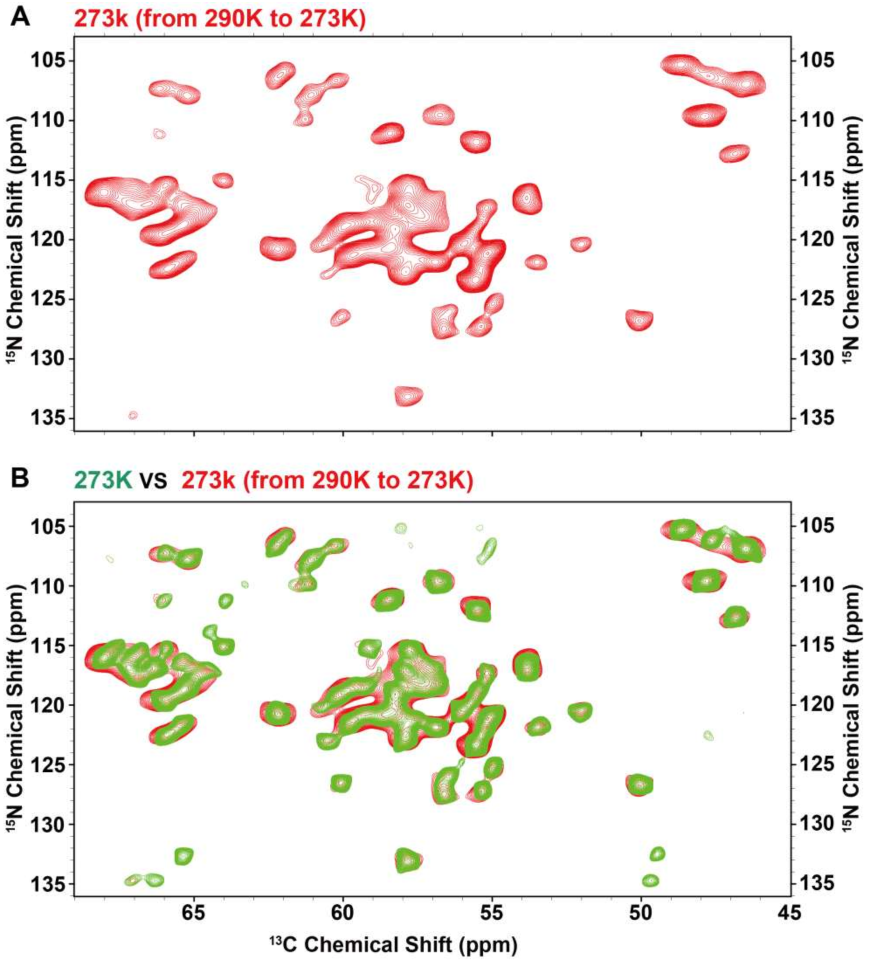

Fig. S13. Reversibility of temperature effects validated by cyclic 2D NCA experiments. (A) To test the reversibility of temperature-induced spectral changes, we performed 2D NCA experiments at 273 K after maintaining the sample at 290 K for 5 days. (B) The post-cycling 273 K spectrum closely matched the initial 273 K reference spectrum (acquired prior to heating), confirming full reversibility of temperature effects.

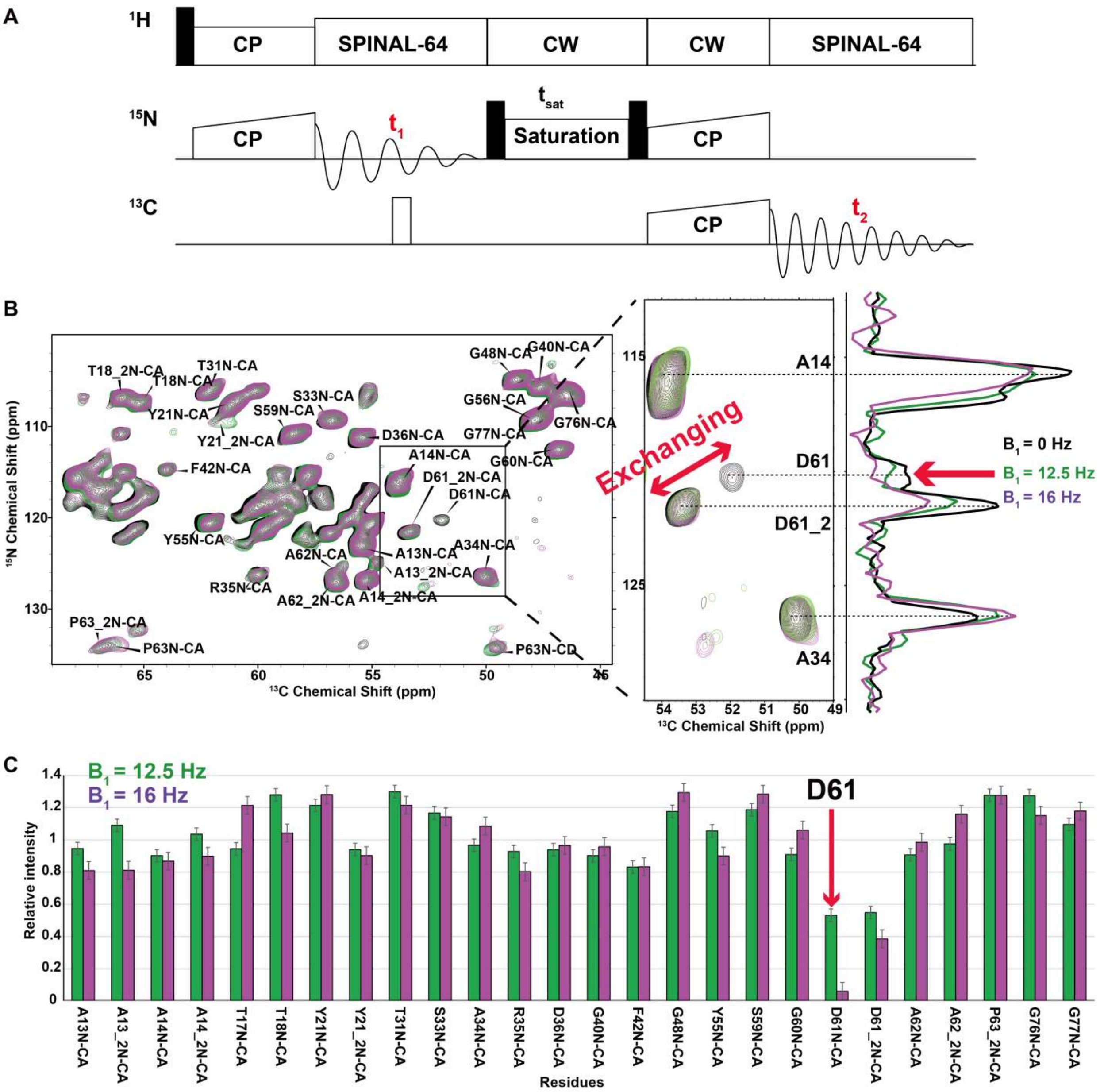

Fig. S14. Conformational exchange between two D61 states probed by 2D CEST-NCA experiments. (A) 2D CEST-NCA pulse sequence. Since the timescale of spin diffusion between ^{13}C nuclei overlaps with that of conformational exchange, we selectively monitored ^{15}N nuclei during exchange. ^{15}N magnetization was generated via cross-polarization (CP), followed by chemical shift encoding in the t1 dimension. Two 90° pulses aligned the ^{15}N magnetization along the Z-axis for chemical exchange. During the exchange period, a weak saturation field (B1= 0–16 Hz in this study) was applied at a specific frequency for a duration Tsat. Subsequently, CP transferred the ^{15}N magnetization to ^{13}C for chemical shift encoding in the t2 dimension(*66*). (B) CEST saturation on D61 conformation 1. To investigate exchange between the two states of residue D61, the CEST irradiation frequency was centered on conformation 1 (120.5 ppm). Saturation power was incrementally increased (0 Hz → 12.5 Hz → 16 Hz; Tsat = 500 ms), and three NCA spectra were recorded at 273K. (C) Signal Intensity Analysis. Signal intensities were normalized to the B_1 = 0 Hz reference spectrum. All isolated peaks were analyzed for intensity variations under different B_1 fields.

.

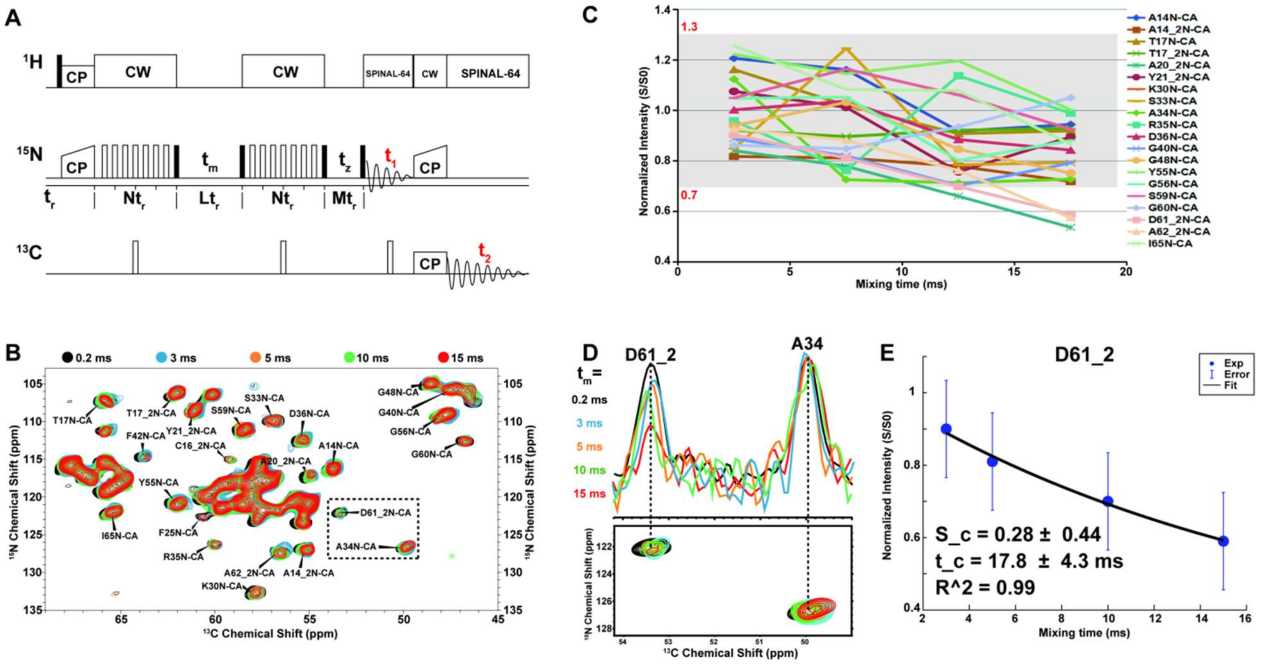

Fig. S15. Quantitative analysis of conformational exchange rates via 2D CSA-CODEX-NCA experiments. (A) 2D CSA-CODEX-NCA pulse sequence. The pulse sequence employs two rotor-synchronized 180°composite pulse blocks (interpulse spacing = half rotor period). The first block recouples ^{15}N anisotropic chemical shifts, inducing signal attenuation, while the second (phase-inverted) block restores the signal. Two separated 90° pulses between the composite blocks generate a stimulated spin echo. A mixing period (tm) is inserted between the 90° pulses. If nuclear spins undergo exchange in anisotropic interactions during tm, incomplete refocusing of phase differences accumulated during chemical shift anisotropy (CSA) recoupling leads to signal attenuation. Contributions from spin diffusion (T1 relaxation) during tm are minimized by introducing a fixed delay (tz) after the second CSA recoupling block. For exchange spectra ($tm \gg tz$) and reference spectra ($tm \ll tz$), the total time (tm+tz) remains constant, ensuring identical T1 effects in both. Normalized exchange signal (S/S_0) reflects pure chemical exchange, and fitting S/S_0 versus tm yields slow exchange parameters(6). (B) 2D CSA-CODEX-NCA spectra at mixing times tm= 0.2, 3, 5, 10, 15 ms were recorded at 290K. (C) Signal intensity changes of 20 independent peaks as a function of tm. Only three residues—A20_2, D61_2, and A62_2—exhibited notable intensity changes ($\Delta I > 0.3$). (D) Spectra of residues D61_2 with notable intensity changes . (E) Correlation times (Tc) were determined as 17.8 ± 4.3 ms by exponential fitting (S/S0 = S_c + (1 - S_c)·exp(t_m/t_c)) (*67*) of the corresponding intensity decays.

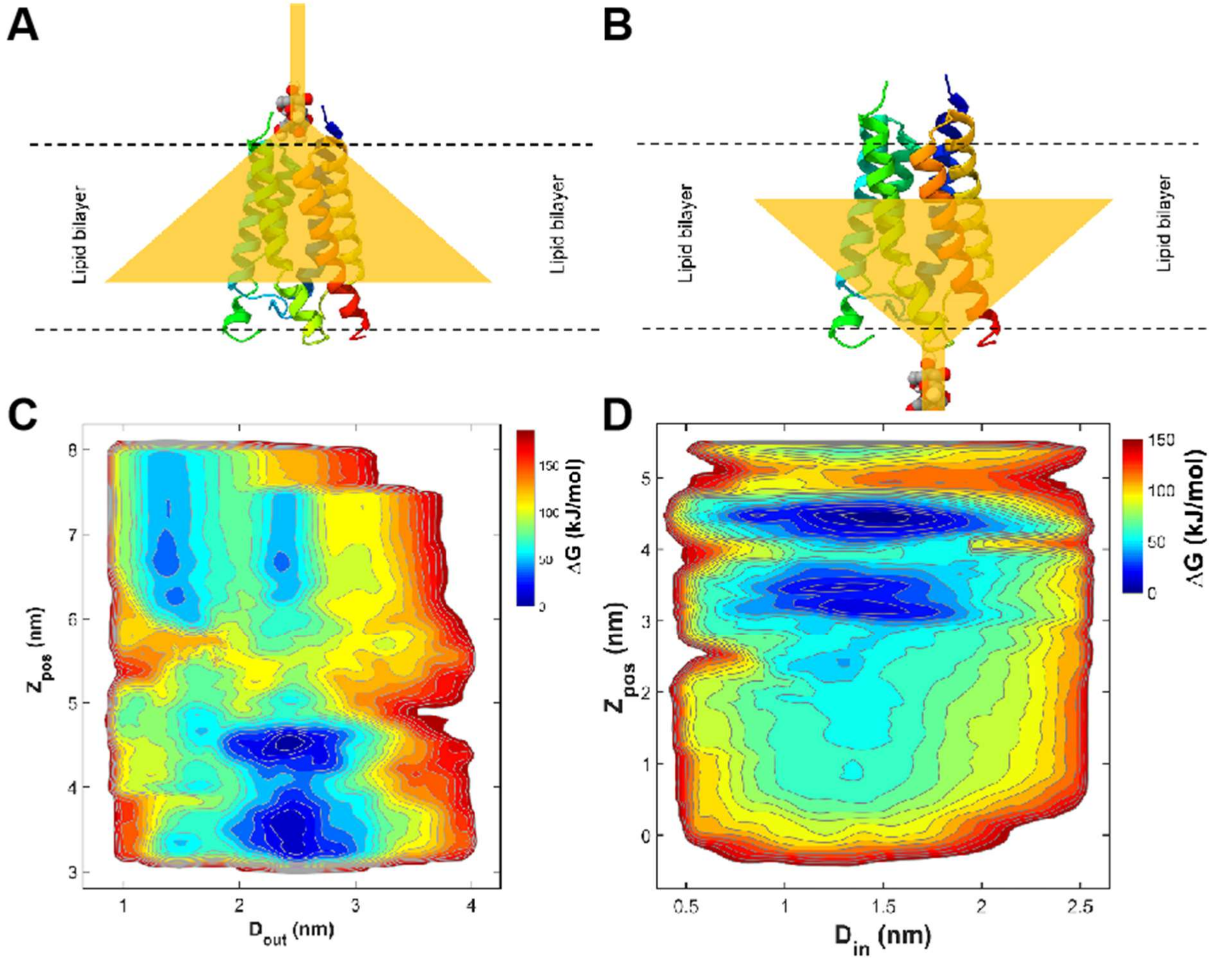

Fig. S16. Funnel metadynamics to study the transporting process of sucrose. (A) Setting of funnel metadynamics (FM) to study the binding process of sucrose. A funnel-style restraint potential was added to make sure the ligands are moving in the restricted region. As an enhanced sampling method, the FM simulation could sample the ligand binding process with high efficiency. The free energy landscapes of the binding process are recovered by FM simulations. **(B)** Setting of funnel metadynamics (FM) to study the releasing process of sucrose. A restraint potential was added near the inner gate region. **(C)** 2D free energy landscapes of sucrose binding process to *Bj*SemiSWEET. The x-axis collective variable (CV) of the free energy landscape is to describe the opening degree of the outward gate, which calculate the distance between the Cα atoms of residue D61 on the two protomer of *Bj*SemiSWEET. Y-axis CV is corresponding to the position of center-of-mass (COM) of sucrose along the direction perpendicular to the membrane interface plane. The low free energy minima in the FELs are colored in blue and high energy regions are colored in red. **(D)** 2D free energy landscapes of sucrose binding process to *Bj*SemiSWEET. The x-axis collective variable (CV) of the free energy landscape is to describe the opening degree of the outward gate, which calculate the distance between the Cα atoms of residue D36 on the two protomer of *Bj*SemiSWEET. Y-axis CV is corresponding to the position of center-of-mass (COM) of sucrose along the direction perpendicular to the membrane interface plane. The low free energy minima in the FELs are colored in blue and high energy regions are colored in red.

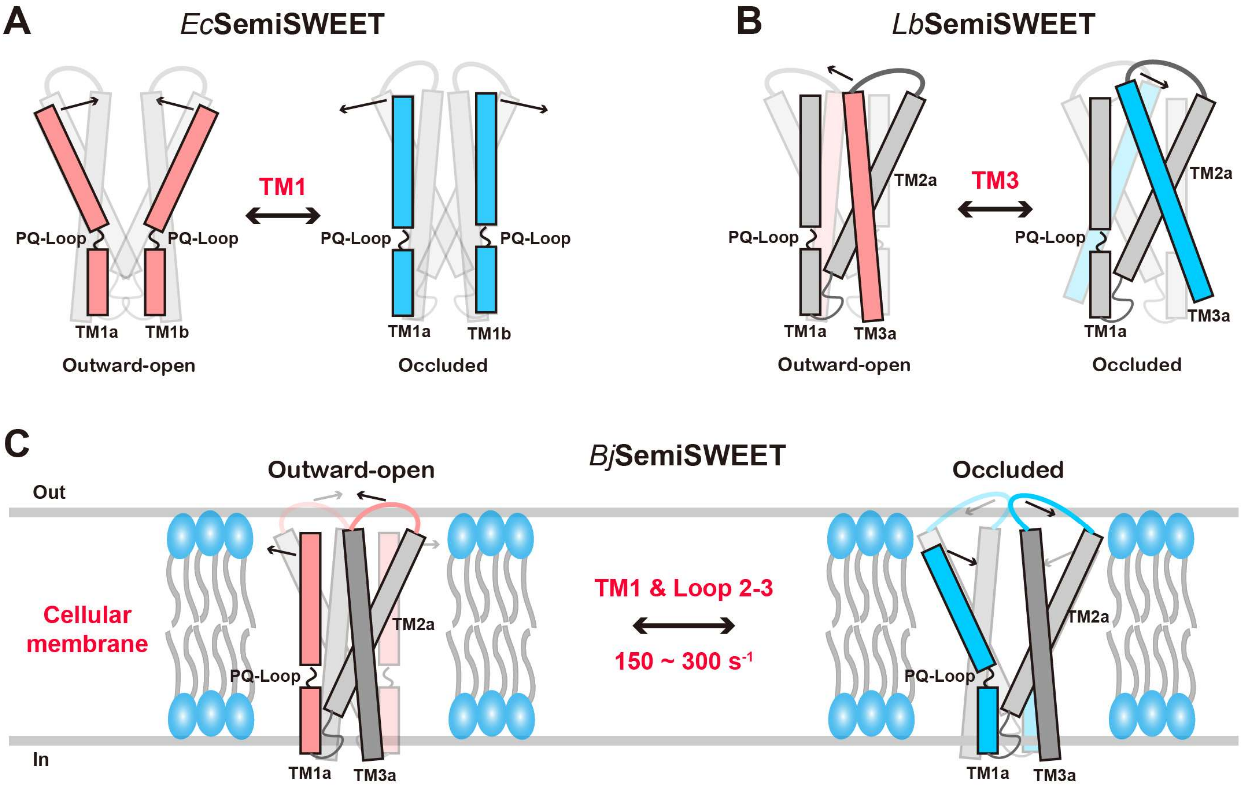

Fig. S17. The conformational transition mechanism of *Bj*SemiSWEET revealed by *in situ* ssNMR in this study differs from those based on X-ray crystallography structures in previous studies. (A) Crystallography analysis revealed the conformational transition mechanism of *Ec*SemiSWEET. By capturing both outward-open and inward-open static structures, it suggested that a hinge-like motion within each protomer, facilitated by the conserved PQ loop, plays a pivotal role. The movement of TM1 contributes to the closure of the extracellular gate. **(B)** Insights from crystallography on the conformational transition mechanism of *Lb*SemiSWEET indicate that rigid body motions within each protomer drive the conformational alterations. Different structures of *Lb*SemiSWEET, including wild-type and mutant forms in occluded, inward, and outward open conformations, show that changes primarily in TM3 lead to the closure of the extracellular gate. **(C)** *In situ* ssNMR has elucidated the conformational transition mechanism of *Bj*SemiSWEET within cellular membranes. It not only elucidated the structures of the outward-open and occluded conformations of *Bj*SemiSWEET in the cellular membrane but also observed rapid inter-conversion between these states on a millisecond to second timescale. Drawing from this array of experimental observations, we present a molecular mechanism delineating the conformational transitions of SemiSWEET within its native cellular membrane environment. When the transmembrane helix 1 (TM1) of a monomer establishes stable contact with Loop L2-3, the structure stabilizes in the outward-open conformation. Conversely, interactions between the Loop L2-3 regions of distinct monomers lead to the structure stabilizing in the occluded conformation. These conformational transitions are primarily instigated by the interplay between the unfavorable conformation of TM1 and electrostatic interactions with the phospholipid headgroups. This proposes mechanism stands in contrast to those posited in prior studies, offering distinctive insights into the mechanism governing the SemiSWEET conformational transition.

Table S1.Quantitative characterization of components in DMPC/DMPG liposomes and native cellular membranes.

Component		Cellular membranes	Synthetic membranes
Total dry weight (mg)		100	100
Total Proteins[a] (mg)	labeled BjSemiSWEET[b]	27.9 ± 3.5	44.44
	labeled background proteins[c]	7.4 ± 0.9	0
	Unlabeled background proteins[d]	26.0 ± 3.3	0
Lipid (mg)		38.7 ± 4.8	55.56
Lipid / BjSemiSWEET protein(w/w)		1.4 ± 0.2	1.25

Detailed methods for measuring protein components in different samples are provided in Methods and the previous study(*26, 37*).

[a]Total protein content in native membranes was quantified by integrating ^{15}N NMR signal intensities of all ^{15}N-labeled proteins,calibrated against the purified BjSemiSWEET sensitivity in the synthetic membranes.

[b]BjSemiSWEET content was determined by normalizing cross-peak intensities in 2D NCA spectra (cellular membranes) to those in DMPC/DMPG liposomes.

[c]Labeled background proteins = Labeled proteins − Labeled BjSemiSWEET. Labeled proteins content in native membranes was quantified by integrating ^{15}N NMR signal intensities of ^{15}N-labeled proteins,calibrated against the purified BjSemiSWEET sensitivity in the synthetic membranes.

[d]Unlabeled proteins = Total proteins − Labeled proteins.

Table S2. Summary of MAS NMR experiments.

ID	Samples	Experiment	^{13}C-^{13}C Mixing (ms)	Field (T)	MAS Rate (kHz)	Experimenta l Time (hrs)
1	U- ^{13}C,^{15}N *Bj*SemiSWEET cellular membrane	2D NCA	-	18.8	10.5	15.4
		2D CORD	50	18.8	10.5	27.6
		2D NcaCX (N_SW@3.5kHz)	50	18.8	10.5	22.4
		2D NcaCX (N_SW@10.5kHz)	50	18.8	10.5	28.2
		2D NcoCX	100	18.8	10.5	25.6
		3D NCACX	50	18.8	10.5	170.7
		3D NCOCX	80	18.8	10.5	85.3
		3D CONCA	20	18.8	10.5	112.6
		3D NCACB	6	18.8	10.5	107.5
		2D CHHC	0.5	18.8	10.5	218.9
		2D PAR	20	18.8	20.0	133.7
2	U- ^{13}C,^{15}N *Bj*SemiSWEET cellular membrane leu rev-label	2D NCA	-	18.8	10.5	14.9
		3D CONCA	20	18.8	10.5	78.2
3	U- ^{13}C,^{15}N *Bj*SemiSWEET cellular membrane C43(DE3) strain	2D NCA	-	18.8	10.5	21.3
4	U- ^{13}C,^{15}N *Bj*SemiSWEET cellular total membrane	2D NCA	-	18.8	10.5	35.8
5	U- ^{13}C,^{15}N *Bj*SemiSWEET POPC/POPG synthetic membrane	2D NCA	-	14.1	8.0	36.4
6	U- ^{13}C,^{15}N *Bj*SemiSWEET DMPC/DMPG synthetic membrane	2D NCA	-	14.1	8.0	32.3
		3D NCACX	50	14.1	8.0	138.9
7	U- ^{13}C,^{15}N *Bj*SemiSWEET cellular membrane various temperature	2D NCA (Temp@273 K)	-	18.8	10.5	15.4
		2D NCA (Temp@283 K)	-	18.8	10.5	15.4
		2D NCA (Temp@285 K)	-	18.8	10.5	15.4
		2D NCA (Temp@288 K)	-	18.8	10.5	15.4
		2D NCA (Temp@290 K)	-	18.8	10.5	15.4
		2D NCA	-	18.8	10.5	15.4

		(Temp@293K)				
		2D CORD (Temp@263 K)	50	18.8	10.5	27.6
		2D CORD (Temp@273 K)	50	18.8	10.5	27.6
		2D CORD (Temp@285 K)	50	18.8	10.5	27.6
8	U- ^{13}C,^{15}N, *Bj*SemiSWEET cellular membrane H/D exchange	2D NCA	-	14.1	8.0	33.9
9	[2-glycero, U-^{15}N] *Bj*SemiSWEET cellular membrane	2D CORD	100	18.8	10.5	45.2
		2D CORD	500	18.8	10.5	163.8
10	[1,3-glycero, U-^{15}N] *Bj*SemiSWEET cellular membrane	2D CORD	100	18.8	10.5	44.8
		2D CORD	500	18.8	10.5	102.4

Table S3. Statistics on the number of distance restraints for the four ssNMR spectra of *Bj*SemiSWEET in cellular membranes by which to assign distance restraints.

Experiment Type	CORD	CORD	CHHC	PAR	Sum
Sample	[2-^{13}C-Glycero, U-^{15}N]	[1,3-^{13}C-Glycero, U-^{15}N]	U-^{13}C,^{15}N	U-^{13}C,^{15}N	
Intra-residue restraints	102	9	17	1	129
Sequential restraints (|i – j| = 1)	97	29	14	5	145
Medium range restraints (2 ≤ |i – j| ≤ 4)	93	39	16	5	153
Long range restraints (|i – j| ≥ 5)	45	5	4	2	56
Inter-monomer restraints	38	1	2	2	43
Sum	375	83	53	15	526

Table S4. Summary of the 16 ambiguous distance restraints of *Bj*SemiSWEET in cellular membranes for CS-Rosetta structure calculation.

Number	w1-^{13}C /ppm	Residue /Atom	w2-^{13}C /ppm	Residue /Atom	Degeneracy	Assignments with CS-Rosetta Structures
1	53.7	A14_1CA	48.4	G48CA	1	A14_1CA-G48CA /Inter-monomer
2	59.7	I37CA/W52CA	53.7	A14_1CA	2	W52CA-A14_1CA /Inter-monomer
3	60.9	Y21_1CA	46.4	G10CA/G76CA	2	Y21_1CA-G76CA /Intra-monomer
4	37.0	I78CB	64.0	F42CA/S59CB	2	I78CB-F42CA /Intra-monomer
5	37.0	I78CB	56.7	A62_2CA/S33CA/A22CA	3	I78CB-A22CA /Intra-monomer
6	61.8	T31CA/S38CA/Y55CA	14.2	I9CD1	3	Y55CA-I9CD1 /Inter-monomer
7	40.8	Y21_1CB/L47CB	64.0	F42CA/S59CB	4	Y21_1CB-F42CA /Inter-monomer
8	59.6	I37CA/W52CA	46.2	G10CA/G76CA	4	W52CA-G10CA /Inter-monomer
9	67.1	V28CA/P63_2CA	46.3	G10CA/G76CA	4	P63_2CA-G10CA /Intra-monomer
10	67.9	T15_1CA/T15_2CA/T18_1CB/T18_2CB	48.4	G48CA	4	T15_1CA-G48CA /Inter-monomer, T15_2CA-G48CA /Inter-monomer
11	66.8	V70CA/P63_2CA/V28CA	59.2	C16_2CA/I37CA	6	V28CA-I37CA /Inter-monomer
12	23.4	L39CD2/V44CG1/L58CD1	61.2	Y21_1CA/Y21_2CA	6	V44CG1-Y21_1CA /Inter-monomer, V44CG1-Y21_2CA /Inter-monomer
13	57.6	Q24CA/L49CA/L74CA	56.1	R32CA/S38CA	6	Q24CA-S38CA /Inter-monomer
14	62.1	T17_2CA/T31CA/S38CB/Y55CA	46.3	G10CA/G76CA	8	Y55CA-G10CA /Inter-monomer
15	66.9	V28CA/P63_2CA/V70CA	47.6	G40CA/G56CA/G77CA	9	P63_2CA-G56CA /Intra-monomer, P63_2CA-G56CA /Inter-monomer
16	56.8	A62_2CA/S33CA/A22CA/L5CA	21.2	T15CG2/V28CG2/T17_2CG2	12	S33CA-V28CG2 /Intra-monomer, S33CA-V28CG2

Table S5. List of long-range distance constraints of *Bj*SemiSWEET in cellular membranes.

Categories	Conformations I			Conformations II		
	Number	Degeneracy	Distance restraints	Number	Degeneracy	Distance restraints
	1	1	D61CA-P3CD	1	1	A62CA-I6CB
	2	1	A62CA-P3CB	2	1	D61CA-K7CB
	3	1	A62CB-P3CA	3	1	D61CA-K7CO
	4	1	D61CA-F4CB	4	1	D61CA-L8CB
	5	1	A62CA-F4CB	5	1	A62CAL8CB
	6	1	P63CA-F4CA	6	1	P63CD-L8CG
	7	1	A62CB-L5CB	7	1	A62CA-I9CB
	8	1	A62CB-L5CA	8	1	G10CA-D61CB
	9	1	P63CA-L5CA	9	1	A62CA-G10CA
	10	1	A62CA-I6CG1	10	1	P63CD-G10CA
	11	1	A62CA-I6CA	11	1	P63CA-G10CA
	12	1	G10CA-L64CB	12	1	P63CA-A12CA
	13	1	N68CA-T15CG2	13	1	C16CA-Y21CB
	14	1	L73CB-T18CB	14	1	A22CA-A75CA
	15	1	Y21CA-A75CA	15	1	I78CB-A22CA
	16	1	A75CA-Y21CB	16	1	P23CD-L78CD1
	17	1	A22CA-A75CA	17	1	V28CA-G77CA
	18	1	I78CB-A22CA	18	1	R35CA-L83CB
	19	1	P23CD-L78CD1	19	1	F42CA-I78CB
	20	1	V28CA-G77CA	20	1	G56CA-I65CD1
	21	1	R35CA-L83CB	21	1	A66CA-D61CA
Intra-Monomer	22	1	F42CA-I78CB	22	1	A66CA-D61CB
	23	1	G56CA-I65CD1	23	2	V46CA-G76CA
	24	2	A12CA-N68CA			I9CA-G10CA
			W52CA-A50CA	24	2	G56CA-N68CB
	25	2	G56CA-N68CB			G56CA-I54CB
			G56CA-I54CB	25	3	S67CA-A14CA
	26	2	V46CA-G76CA			T15CB-A14CA
			I9CA-G10CA			T18CB-A14CA
	27	3	F42CA-I78CA	26	3	N68CA-A14CA
			V44CA-F42CA			N68CA-A12CA
			V46CA-F42CA			W52CA-A50CA
	28	3	G56CA-N68CA	27	3	I78CA-F42CA
			W52CA-G56CA			V44CA-F42CA
			I37CA-G40CA			V46CA-F42CA
	29	4	A14CA-S67CA	28	3	N68CA-G56CA
			T15CB-A14CA			W52CA-G56CA
			T18CB-A14CA			I37CA-G40CA
			C16CA-A14CA	29	4	T15CB-N68CA
	30	4	T15CB-N68CA			I65CA-N68CA
			I65CA-N68CA			T71CA-N68CA
			T71CA-N68CA			S67CA-N68CA
			S67CA-N68CA			
	1	1	S59CB-I6CB	1	1	S59CB-I6CB
Inter-Monomer	2	1	Y55CA-I9CD1	2	1	Y55CA-I9CD1
	3	1	G10CA-L51CD1	3	1	G10CA-L51CD1

4	1	G10CA-L51CG	4	1	G10CA-L51CG
5	1	G10CA-W52CB	5	1	G10CA-W52CB
6	1	W52CA-G10CA	6	1	W52CA-G10CA
7	1	Y55CA-G10CA	7	1	Y55CA-G10CA
8	1	G10CA-L58CD1	8	1	G10CA-L58CD1
9	1	G10CA-L58CD2	9	1	G10CA-L58CD2
10	1	A14CA-L47CB	10	1	L47CA-A14CA
11	1	A14CA-G48CA	11	1	A14CA-G48CA
12	1	W52CA-A14CA	12	1	A14CA-L51CG
13	1	F42CA-A20CA	13	1	W52CA-A14CA
14	1	F42CA-Y21CB	14	1	A14CA-W52CB
15	1	V44CG1-Y21CA	15	1	G48CA-T15CG2
16	1	Y21CA-M45CA	16	1	F42CA-A20CA
17	1	V28CA-I37CA	17	1	F42CA-Y21CB
18	1	Q24CA-S38CA	18	1	Y21CA-V44CG1
19	2	G48CA-T15CG2	19	1	Y21CA-M45CA
		G48CA-V46CG2	20	1	V28CA-I37CA
20	2	T15CA-G48CA	21	1	Q24CA-S38CA
		T18CB-G48CA	22	1	P63CA-W52CA
21	2	T15CA-V44CG1	23	1	A62CB-G56CA
		T18CB-V44CG1	24	1	G56CA-D61CB
22	2	T17CA-V44CA	25	1	P63CA-L57CD1
		I54CA-Y55CA	26	1	P63CA-L58CD1
23	2	S33CA-V28CG2	27	1	D61CA-L58CG
		intra,S33CA-V28CG2	28	2	T15CA-G48CA
24	2	S33CA-A34CA			T18CB-G48CA
		intra,S33CA-A34CA	29	2	T15CB-G48CA
25	2	A34CA-S33CB			T18CA-G48CA
		intra,A34CA-S33CB	30	2	T15CA-V44CG1
26	4	V28CA-D36CB			T18CB-V44CG1
		V28CA-K30CE	31	2	C16CA-L47CB
		V28CA-K27CE			C16CA-Y21CB
		V70CA-L74CB	32	2	C16CA-G48CA
					W52CA-G48CA
			33	2	S33CA-V28CG2
					intra,S33CA-V28CG2
			34	2	P63CA-G56CA
					intra,P63CA-G56CA
			35	3	M45CA-T17CA
					L47CA-T17CA
					Y55CA-S59CA
			36	5	V28CA-D36CB
					V28CA-K30CE
					V28CA-K27CE
					P63CA-D61CB
					V70CA-L74CB

Table S6. Statistics for structure determination of *Bj*SemiSWEET in cellular membranes.

	Outward-open state	Occluded state
	BMRB ID: 36702, PDB ID: 9KAX	BMRB ID: 36703, PDB ID: 9KBA
**NMR distance and dihedral constraints**		
**Distance constraints**		
**Total NOE**	430 × 2	419 × 2
**Intra-residue**	85 × 2	75 × 2
**Inter-residue**	271 × 2	269 × 2
**Sequential (|i – j| = 1)**	112 × 2	98 × 2
**Medium range (2 ≤ |i – j| ≤ 4)**	103 × 2	106 × 2
**Long range (|i – j| ≥ 5)**	30 × 2	29 × 2
**Intermolecular**	26 × 2	36 × 2
**Hydrogen bonds**	74 × 2	75 × 2
**Total dihedral-angle restraints**		
**ϕ**	79 × 2	79 × 2
**ψ**	79 × 2	79 × 2
**Structure statistics**		
**Violations (mean ± s.d.)**		
**Distance constraints (Å)**	0.003 ± 0.002	0.004 ± 0.002
**Dihedral-angle constraints (°)**	1.466 ± 0.182	1.250 ± 0.176
**Max dihedral-angle violation (°)**	8.94	10.88
**Max distance-constraint violation (Å)**	0.98	2.15
**Deviations from idealized geometry**		
**bond lengths (Å)**	0.003 ± 0.001	0.004 ± 0.001
**bond angles (°)**	0.457 ± 0.013	0.473 ± 0.025
**Impropers (°)**	0.354 ± 0.015	0.375 ± 0.034
**Average pairwise r.m.s.d. (Å)[a] 0**		
**Heavy**	2.2 ± 0.2	3.2 ± 0.3
**backbone**	1.5 ± 0.2	2.5 ± 0.3
**Structural quality**		
**Ramachandran Plot Statistics [b]**		
**Residues in most favored region (%)**	98.6	97.3
**Residues in additional allowed region (%)**	0.0	2.7
**Residues in generously allowed region (%)**	0.0	0.0
**Residues in disallowed region (%)**	1.4	0.0
**Clashscore[c]**	8.54	6.31
**Molprobity score (Å)**	1.46	1.35

[a]Pairwise r.m.s. deviation was calculated between 10 lowest energy structures.
[b]Evaluated with the program PROCHECK(*63*).
[c]Evaluated with the Molprobity program(*64*).

REFERENCES AND NOTES

1. D. Drew, R. A. North, K. Nagarathinam, M. Tanabe, Structures and general transport mechanisms by the major facilitator superfamily (MFS). *Chem. Rev.* **121**, 5289–5335 (2021).

2. A. César-Razquin, B. Snijder, T. Frappier-Brinton, R. Isserlin, G. Gyimesi, X. Bai, R. A. Reithmeier, D. Hepworth, M. A. Hediger, A. M. Edwards, G. Superti-Furga, A call for systematic research on solute carriers. *Cell* **162**, 478–487 (2015).

3. N. R. Latorraca, N. M. Fastman, A. J. Venkatakrishnan, W. B. Frommer, R. O. Dror, L. Feng, Mechanism of substrate translocation in an alternating access transporter. *Cell* **169**, 96–107.e12 (2017).

4. L. Feng, W. B. Frommer, Structure and function of SemiSWEET and SWEET sugar transporters. *Trends Biochem. Sci.* **40**, 480–486 (2015).

5. M. Ernst, J. L. Robertson, the role of the membrane in transporter folding and activity. *J. Mol. Biol.* **433**, 167103 (2021).

6. C. Martens, R. A. Stein, M. Masureel, A. Roth, S. Mishra, R. Dawaliby, A. Konijnenberg, F. Sobott, C. Govaerts, H. S. McHaourab, Lipids modulate the conformational dynamics of a secondary multidrug transporter. *Nat. Struct. Mol. Biol.* **23**, 744–751 (2016).

7. S. Narasimhan, G. E. Folkers, M. Baldus, When small becomes too big: Expanding the use of in-cell solid-state NMR spectroscopy. *ChemPlusChem* **85**, 760–768 (2020).

8. S. Wu, A. Avila-Sakar, J. M. Kim, D. S. Booth, C. H. Greenberg, A. Rossi, M. Liao, X. Li, A. Alian, S. L. Griner, N. Juge, Y. Yu, C. M. Mergel, J. Chaparro-Riggers, P. Strop, R. Tampé, R. H. Edwards, R. M. Stroud, C. S. Craik, Y. Cheng, Fabs Enable single particle cryoEM studies of small proteins. *Structure* **20**, 582–592 (2012).

9. T. Uchański, E. Pardon, J. Steyaert, Nanobodies to study protein conformational states. *Curr. Opin. Struct. Biol.* **60**, 117–123 (2020).

10. B. C. McIlwain, A. L. Erwin, A. R. Davis, B. Ben Koff, L. Chang, T. Bylund, G.-Y. Chuang, P. D. Kwong, M. D. Ohi, Y.-T. Lai, R. B. Stockbridge, N-terminal transmembrane-helix epitope tag for x-ray crystallography and electron microscopy of small membrane proteins. *J. Mol. Biol.* **433**, 166909 (2021).

11. T. O. Yeates, M. P. Agdanowski, Y. Liu, Development of imaging scaffolds for cryo-electron microscopy. *Curr. Opin. Struct. Biol.* **60**, 142–149 (2020).

12. J. J. Ruprecht, M. S. King, T. Zögg, A. A. Aleksandrova, E. Pardon, P. G. Crichton, J. Steyaert, E. R. S. Kunji, The molecular mechanism of transport by the mitochondrial ADP/ATP carrier. *Cell* **176**, 435–447.e15 (2019).

13. D. Drew, O. Boudker, Shared molecular mechanisms of membrane transporters. *Annu. Rev. Biophys.* **85**, 543–572 (2016).

14. T. R. Alderson, L. E. Kay, NMR spectroscopy captures the essential role of dynamics in regulating biomolecular function. *Cell* **184**, 577–595 (2021).

15. H. Tan, M. Duan, H. Xie, Y. Zhao, H. Liu, M. Yang, M. Liu, J. Yang, Fast collective motions of backbone in transmembrane α helices are critical to water transfer of aquaporin. *Sci. Adv.* **10**, eade9520 (2024).

16. E. A. Morrison, G. T. DeKoster, S. Dutta, R. Vafabakhsh, M. W. Clarkson, A. Bahl, D. Kern, T. Ha, K. A. Henzler-Wildman, Antiparallel EmrE exports drugs by exchanging between asymmetric structures. *Nature* **481**, 45–50 (2012).

17. V. S. Mandala, M. D. Gelenter, M. Hong, Transport-relevant protein conformational dynamics and water dynamics on multiple time scales in an archetypal proton channel: Insights from solid-state NMR. *J. Am. Chem. Soc.* **140**, 1514–1524 (2018).

18. H. Tan, W. Zhao, M. Duan, Y. Zhao, Y. Zhang, H. Xie, Q. Tong, J. Yang, Native cellular membranes facilitate channel activity of MscL by enhancing slow collective motions of its transmembrane helices. *J. Am. Chem. Soc.* **146**, 31472–31485 (2024).

19. E. Lerner, T. Cordes, A. Ingargiola, Y. Alhadid, S. Chung, X. Michalet, S. Weiss, Toward dynamic structural biology: Two decades of single-molecule Förster resonance energy transfer. *Science* **359**, eaan1133 (2018).

20. T. A. Cross, M. Sharma, M. Yi, H.-X. Zhou, Influence of solubilizing environments on membrane protein structures. *Trends Biochem. Sci.* **36**, 117–125 (2011).

21. K. K. Frederick, V. K. Michaelis, B. Corzilius, T. C. Ong, A. C. Jacavone, R. G. Griffin, S. Lindquist, Sensitivity-enhanced NMR reveals alterations in protein structure by cellular milieus. *Cell* **163**, 620–628 (2015).

22. Y. Zhao, H. Xie, L. Wang, Y. Shen, W. Chen, B. Song, Z. Zhang, A. Zheng, Q. Lin, R. Fu, J. Wang, J. Yang, Gating mechanism of aquaporin Z in synthetic bilayers and native membranes revealed by solid-state NMR spectroscopy. *J. Am. Chem. Soc.* **140**, 7885–7895 (2018).

23. C. Martens, M. Shekhar, A. J. Borysik, A. M. Lau, E. Reading, E. Tajkhorshid, P. J. Booth, A. Politis, Direct protein-lipid interactions shape the conformational landscape of secondary transporters. *Nat. Commun.* **9**, 4151 (2018).

24. R. Jia, C. Martens, M. Shekhar, S. Pant, G. A. Pellowe, A. M. Lau, H. E. Findlay, N. J. Harris, E. Tajkhorshid, P. J. Booth, A. Politis, Hydrogen-deuterium exchange mass spectrometry captures distinct dynamics upon substrate and inhibitor binding to a transporter. *Nat. Commun.* **11**, 6162 (2020).

25. H. X. Zhou, T. A. Cross, Influences of membrane mimetic environments on membrane protein structures. *Annu. Rev. Biophys.* **42**, 361–392 (2013).

26. H. Xie, Y. Zhao, W. Zhao, Y. Chen, M. Liu, J. Yang, Solid-state NMR structure determination of a membrane protein in E. coli cellular inner membrane. *Sci. Adv.* **9**, eadh4168 (2023).

27. M. Renault, R. Tommassen-van Boxtel, M. P. Bos, J. A. Post, J. Tommassen, M. Baldus, Cellular solid-state nuclear magnetic resonance spectroscopy. *Proc. Natl. Acad. Sci. U.S.A.* **109**, 4863–4868 (2012).

28. V. A. Higman, K. Varga, L. Aslimovska, P. J. Judge, L. J. Sperling, C. M. Rienstra, A. Watts, The conformation of bacteriorhodopsin loops in purple membranes resolved by solid-state MAS NMR spectroscopy. *Angew. Chem. Int. Ed. Engl.* **50**, 8432–8435 (2011).

29. R. Fu, X. Wang, C. Li, A. N. Santiago-Miranda, G. J. Pielak, F. Tian, In situ structural characterization of a recombinant protein in native Escherichia coli membranes with solid-state magic-angle-spinning NMR. *J. Am. Chem. Soc.* **133**, 12370–12373 (2011).

30. Y. Miao, H. Qin, R. Fu, M. Sharma, T. V. Can, I. Hung, S. Luca, P. L. Gor'kov, W. W. Brey, T. A. Cross, M2 proton channel structural validation from full-length protein samples in synthetic bilayers and E. coli membranes. *Angew. Chem. Int. Ed. Engl.* **51**, 8383–8386 (2012).

31. T. Jacso, W. T. Franks, H. Rose, U. Fink, J. Broecker, S. Keller, H. Oschkinat, B. Reif, Characterization of membrane proteins in isolated native cellular membranes by dynamic nuclear polarization solid-state NMR spectroscopy without purification and reconstitution. *Angew. Chem. Int. Ed. Engl.* **51**, 432–435 (2012).

32. N. V. Kulminskaya, M. Pedersen, M. Bjerring, J. Underhaug, M. Miller, N. U. Frigaard, J. T. Nielsen, N. C. Nielsen, In situ solid-state NMR spectroscopy of protein in heterogeneous membranes: The baseplate antenna complex of Chlorobaculum tepidum. *Angew. Chem. Int. Ed. Engl.* **51**, 6891–6895 (2012).

33. M. E. Ward, S. Wang, R. Munro, E. Ritz, I. Hung, P. L. Gor'kov, Y. Jiang, H. Liang, L. S. Brown, V. Ladizhansky, In situ structural studies of Anabaena sensory rhodopsin in the E. coli membrane. *Biophys. J.* **108**, 1683–1696 (2015).

34. S. A. Shahid, M. Nagaraj, N. Chauhan, T. W. Franks, B. Bardiaux, M. Habeck, M. Orwick-Rydmark, D. Linke, B. J. van Rossum, Solid-state NMR study of the YadA membrane-anchor domain in the bacterial outer membrane. *Angew. Chem. Int. Ed. Engl.* **54**, 12602–12606 (2015).

35. C. Sun, X. Ding, H. Cui, Y. Yang, S. Chen, A. Watts, X. Zhao, In situ study of the function of bacterioruberin in the dual-chromophore photoreceptor archaerhodopsin-4. *Angew. Chem. Int. Ed.* **57**, 8937–8941 (2018).

36. R. Shukla, A. J. Peoples, K. C. Ludwig, S. Maity, M. G. N. Derks, S. De Benedetti, A. M. Krueger, B. J. A. Vermeulen, T. Harbig, F. Lavore, R. Kumar, R. V. Honorato, F. Grein, K. Nieselt, Y. Liu, A. M. J. J. Bonvin, M. Baldus, U. Kubitscheck, E. Breukink, C. Achorn, A. Nitti, C. J. Schwalen, A. L. Spoering, L. L. Ling, D. Hughes, M. Lelli, W. H. Roos, K. Lewis, T. Schneider, M. Weingarth, An antibiotic from an uncultured bacterium binds to an immutable target. *Cell* **186**, 4059–4073.e27 (2023).

37. Y. Zhang, Y. Gan, W. Zhao, X. Zhang, Y. Zhao, H. Xie, J. Yang, Membrane protein structures in native cellular membranes revealed by solid-state NMR spectroscopy. *JACS Au* **3**, 3412–3423 (2023).

38. Y. H. Xuan, Y. B. Hu, L.-Q. Chen, D. Sosso, D. C. Ducat, B.-H. Hou, W. B. Frommer, Functional role of oligomerization for bacterial and plant SWEET sugar transporter family. *Proc. Natl. Acad. Sci. U.S.A.* **110**, E3685–E3694 (2013).

39. J. Wang, C. Yan, Y. Li, K. Hirata, M. Yamamoto, N. Yan, Q. Hu, Crystal structure of a bacterial homologue of SWEET transporters. *Cell Res.* **24**, 1486–1489 (2014).

40. Y. Xu, Y. Tao, L. S. Cheung, C. Fan, L.-Q. Chen, S. Xu, K. Perry, W. B. Frommer, L. Feng, Structures of bacterial homologues of SWEET transporters in two distinct conformations. *Nature* **515**, 448–452 (2014).

41. Y. Lee, T. Nishizawa, K. Yamashita, R. Ishitani, O. Nureki, Structural Basis for the Facilitative Diffusion Mechanism by SemiSWEET Transporter. *Nat. Commun.* **6**, 6112 (2015).

42. G. Hou, S. Yan, J. Trébosc, J. P. Amoureux, T. Polenova, Broadband homonuclear correlation spectroscopy driven by combined $R2_n^v$ sequences under fast magic angle spinning for NMR structural analysis of organic and biological solids. *J. Magn. Reson.* **232**, 18–30 (2013).

43. F. Castellani, B. van Rossum, A. Diehl, M. Schubert, K. Rehbein, H. Oschkinat, Structure of a protein determined by solid-state magic-angle-spinning NMR spectroscopy. *Nature* **420**, 99–102 (2002).

44. G. De Paëpe, J. R. Lewandowski, A. Loquet, A. Böckmann, R. G. Griffin, Proton assisted recoupling and protein structure determination. *J. Chem. Phys.* **129**, 245101 (2008).

45. A. Lange, S. Luca, M. Baldus, Structural constraints from proton-mediated rare-spin correlation spectroscopy in rotating solids. *J. Am. Chem. Soc.* **124**, 9704–9705 (2002).

46. Y. Shen, F. Delaglio, G. Cornilescu, A. Bax, TALOS+: A hybrid method for predicting protein backbone torsion angles from NMR chemical shifts. *J. Biomol. NMR* **44**, 213–223 (2009).

47. I. K. McDonald, J. M. Thornton, Satisfying hydrogen bonding potential in proteins. *J. Mol. Biol.* **238**, 777–793 (1994).

48. C. D. Schwieters, J. J. Kuszewski, N. Tjandra, G. M. Clore, The Xplor-NIH NMR molecular structure determination package. *J. Magn. Reson.* **160**, 65–73 (2003).

49. D. Sehnal, R. Svobodová Vařeková, K. Berka, L. Pravda, V. Navrátilová, P. Banáš, C.-M. Ionescu, M. Otyepka, J. Koča, MOLE 2.0: Advanced approach for analysis of biomacromolecular channels. *J. Chem.* **5**, 39 (2013).

50. B. D. Rao, Nuclear magnetic resonance line-shape analysis and determination of exchange rates. *Meth. Enzymol* **176**, 279–311 (1989).

51. M. H. Levitt, *Spin Dynamics: Basics of Nuclear Magnetic Resonance* (Wiley, 2013).

52. S. Morein, A.-S. Andersson, L. Rilfors, G. Lindblom, Wild-type Escherichia coli cells regulate the membrane lipid composition in a window between gel and non-lamellar structures. *J. Biol. Chem.* **271**, 6801–6809 (1996).

53. E. Papaleo, G. Saladino, M. Lambrughi, K. Lindorff-Larsen, F. L. Gervasio, R. Nussinov, The role of protein loops and linkers in conformational dynamics and allostery. *Chem. Rev.* **116**, 6391–6423 (2016).

54. S. Wang, R. A. Munro, L. Shi, I. Kawamura, T. Okitsu, A. Wada, S. Y. Kim, K. H. Jung, L. S. Brown, V. Ladizhansky, Solid-state NMR spectroscopy structure determination of a lipid-embedded heptahelical membrane protein. *Nat. Methods* **10**, 1007–1012 (2013).

55. F. X. Theillet, A. Binolfi, B. Bekei, A. Martorana, H. M. Rose, M. Stuiver, S. Verzini, D. Lorenz, M. van Rossum, D. Goldfarb, P. Selenko, Structural disorder of monomeric α-synuclein persists in mammalian cells. *Nature* **530**, 45–50 (2016).

56. F. K. Schur, Toward high-resolution in situ structural biology with cryo-electron tomography and subtomogram averaging. *Curr. Opin. Struct. Biol.* **58**, 1–9 (2019).

57. M. Baldus, A. T. Petkova, J. Herzfeld, R. G. Griffin, Cross polarization in the tilted frame: Assignment and spectral simplification in heteronuclear spin systems. *Mol. Phys.* **95**, 1197–1207 (1998).

58. K. Takegoshi, S. Nakamura, T. Terao, ^{13}C–^{1}H dipolar-assisted rotational resonance in magic-angle spinning NMR. *Chem. Phys. Lett.* **344**, 631–637 (2001).

59. F. Delaglio, S. Grzesiek, G. W. Vuister, G. Zhu, J. Pfeifer, A. Bax, NMRPipe: A multidimensional spectral processing system based on UNIX pipes. *J. Biomol. NMR* **6**, 277–293 (1995).

60. D. S. Wishart, B. D. Sykes, F. M. Richards, The chemical shift index: A fast and simple method for the assignment of protein secondary structure through NMR spectroscopy. *Biochemistry* **31**, 1647–1651 (1992).

61. Y. Shen, O. Lange, F. Delaglio, P. Rossi, J. M. Aramini, G. Liu, A. Eletsky, Y. Wu, K. K. Singarapu, A. Lemak, A. Ignatchenko, C. H. Arrowsmith, T. Szyperski, G. T. Montelione, D. Baker, A. Bax, Consistent blind protein structure generation from NMR chemical shift data. *Proc. Nat. Acad. Sci. U.S.A.* **105**, 4685–4690 (2008).

62. Y. Shen, A. Bax, Homology modeling of larger proteins guided by chemical shifts. *Nat. Methods* **12**, 747–750 (2015).

63. R. A. Laskowski, M. W. MacArthur, D. S. Moss, J. M. Thornton, PROCHECK: A program to check the stereochemical quality of protein structures. *J. Appl. Cryst.* **26**, 283–291 (1993).

64. V. B. Chen, W. B. Arendall III, J. J. Headd, D. A. Keedy, R. M. Immormino, G. J. Kapral, L. W. Murray, J. S. Richardson, D. C. Richardson, MolProbity: All-atom structure validation for macromolecular crystallography. *Acta Crystallogr. D Biol. Crystallogr.* **66**, 12–21 (2010).

65. M. Merezhko, E. Pakarinen, R. L. Uronen, H. J. Huttunen, Live-cell monitoring of protein localization to membrane rafts using protein-fragment complementation. *Biosci. Rep.* **40**, BSR20191290 (2020).

66. P. Rovó, R. Linser, Microsecond timescale protein dynamics: A combined solid-state NMR approach. *ChemPhysChem* **19**, 34–39 (2018).

67. P. Schanda, M. Ernst, Studying dynamics by magic-angle spinning solid-state NMR spectroscopy: Principles and applications to biomolecules. *Prog. Nucl. Magn. Reson. Spectrosc.* **96**, 1–46 (2016).
